# The role of treatment timing and mode of stimulation in the treatment of primary dysmenorrhea with acupuncture: An exploratory randomised controlled trial

**DOI:** 10.1371/journal.pone.0180177

**Published:** 2017-07-12

**Authors:** Mike Armour, Hannah G. Dahlen, Xiaoshu Zhu, Cindy Farquhar, Caroline A. Smith

**Affiliations:** 1 The National Institute of Complementary Medicine, Western Sydney University, Sydney, Australia; 2 School of Nursing and Midwifery, Western Sydney University, Sydney, Australia; 3 Department of Obstetrics and Gynaecology, University of Auckland, Auckland, New Zealand; Johns Hopkins University Bloomberg School of Public Health, UNITED STATES

## Abstract

**Objectives:**

We examined the effect of changing treatment timing and the use of manual, electro acupuncture on the symptoms of primary dysmenorrhea.

**Methods:**

A randomised controlled trial was performed with four arms, low frequency manual acupuncture (LF-MA), high frequency manual acupuncture (HF-MA), low frequency electro acupuncture (LF-EA) and high frequency electro acupuncture (HF-EA). A manualised trial protocol was used to allow differentiation and individualized treatment over three months. A total of 74 women were randomly assigned to one of the four groups (LF-MA n = 19, HF-MA n = 18, LF-EA n = 18, HF-EA n = 19). Twelve treatments were performed over three menstrual cycles, either once per week (LF groups) or three times in the week prior to menses (HF groups). All groups received a treatment in the first 48 hours of menses. The primary outcome was the reduction in peak menstrual pain at 12 months from trial entry.

**Results:**

During the treatment period and nine month follow-up all groups showed statistically significant (p < .001) reductions in peak and average menstrual pain compared to baseline but there were no differences between groups (p > 0.05). Health related quality of life increased significantly in six domains in groups having high frequency of treatment compared to two domains in low frequency groups. Manual acupuncture groups required less analgesic medication than electro-acupuncture groups (p = 0.02). HF-MA was most effective in reducing secondary menstrual symptoms compared to both–EA groups (p<0.05).

**Conclusion:**

Acupuncture treatment reduced menstrual pain intensity and duration after three months of treatment and this was sustained for up to one year after trial entry. The effect of changing mode of stimulation or frequency of treatment on menstrual pain was not significant. This may be due to a lack of power. The role of acupuncture stimulation on menstrual pain needs to be investigated in appropriately powered randomised controlled trials.

## Introduction

Primary dysmenorrhea is defined as menstrual pain in the absence of any organic cause and is most common in women under the age of 25. Primary dysmenorrhea’s characteristic symptom is crampy, colicky spasms of pain in the suprapubic area, occurring within 8–72 hours of menstruation and peaking within the first few days as menstrual flow increases[1]. In addition to painful cramps, many women with primary dysmenorrhea experience other menstrual-related symptoms, including back and thigh pain, headaches, diarrhoea, nausea and vomiting[1]. Pain commonly starts within three years of menarche[2] and results in life time world-wide prevalence rates ranging from 17–81%[3] with 71% of Australian women suffering from dysmenorrhea in a given year[4]. Primary dysmenorrhea is the most common cause for school absenteeism in women [5] and in 10–30% of women causes regular absence from work, school or university each month [6, 7]. In addition to absenteeism primary dysmenorrhea causes a reduction in academic performance [8, 9], reduced participation in sport and social activities[8] and an overall significant decrease in women’s quality of life [9, 10]. Despite the significant negative impact and disruption to daily living that primary dysmenorrhea has on women most do not seek medical treatment [11, 12].When women do present to their doctor with primary dysmenorrhea, the most commonly prescribed treatments are non-steroidal anti-inflammatories (NSAIDs) and the combined oral contraceptive (COC) pill. These treatments are effective for many women [2, 13], however approximately 25% of women have pain that is refractory to either or both of these standard treatments[14].

Lack of satisfaction in standard treatment leads to an increase in self-care [15, 16], with women commonly using complementary therapies to deal with their menstrual pain[17] in addition to, or instead of, pharmaceutical pain relief, due to a lack of perceived effectiveness[15, 18, 19] or a dislike of using analgesic medication[20]. Surveys of practice show women are presenting to acupuncturists in the community with gynaecological complaints such as primary dysmenorrhea [21] despite the evidence for acupuncture’s effectiveness in treating primary dysmenorrhea being uncertain[22]. Many clinical trials are characterised as having a high risk of bias or a lack of clinical validity due to a significant departure from how acupuncture is delivered in contemporary clinical practice.

Despite the lack of high quality evidence there are plausible mechanisms of action for acupuncture to improve primary dysmenorrhea, including endogenous opioid release[23], reduction of inflammation [24], alterations in uterine blood flow[25] and changes in prostaglandin levels [26]. Acupuncture appears to show a dose response relationship between various needling parameters and pain [27]. There is some evidence that electro-acupuncture provides a more rapid reduction in menstrual pain compared to manual acupuncture; however this finding was based on a single study that used an uncommon style of indwelling acupuncture administered only during menses and assessed using non-validated outcome measures [28]. The timing of treatment around menses has been examined in two studies [29, 30] which suggested that treatment three to seven days prior to menses was more effective than treatment during menses. No previous trials have directly investigated the possible dose response relationship that appears to be present between the use of electro-acupuncture and manual acupuncture or in changing frequency of treatment in a western clinical setting where use of manual acupuncture delivered once per week is more common.

This exploratory study aims to examine the possible impact of changing the mode of acupuncture stimulation and treatment timing on menstrual pain. We hypothesised that both electro-acupuncture and higher frequency of treatment would deliver greater reductions in menstrual pain and greater improvements in health-related quality of life compared to lower frequency or manual acupuncture.

## Materials and methods

### Study design

An exploratory study using a 2x2 factorial design, to test the individual and combined effects of changing 1) treatment timing, and 2) mode of stimulation. This study used a pragmatic clinical trial design with some qualifications, including the use of a manualised acupuncture protocol designed to reduce the amount of variation between practitioners (S1 File). This design allows for a balance between removing excessive inter-practitioner variation and still encompassing the complex nature of traditional Chinese medicine (TCM) clinical practice [31]. When evaluating a complex intervention or “package of care,” such as TCM acupuncture, a design slanted towards the pragmatic effectiveness end of the continuum is more appropriate and increases the ecological validity of the outcome [32]. Acupuncture-specific reporting in this trial follows the revised Standards for Reporting Interventions in Clinical Trials of Acupuncture [33].

The University of Western Sydney Human Ethics Committee (H10082) and the Health and Disability Ethics Committee New Zealand (13/CEN/60) approved this study. Maori approval was obtained from the Office of Tumuaki along with locality assessments for the clinic sites in Auckland and Wellington through the Health and Disability Ethics Committee New Zealand. Written consent was obtained from all participants before trial entry. The trial was prospectively registered with the Australian New Zealand Clinical Trials Registry (https://www.anzctr.org.au/Trial/Registration/TrialReview.aspx?id=363933) ACTRN12613000351718. Recruitment ran from June 2013 to January 2014.

### Participants

Eligible participants were aged 18–45 years, with suspected or confirmed primary dysmenorrhea as defined by the following: a history of period pain beginning before the age of 18, or period pain beginning after the age of 18, but gynaecological investigations by laparoscopy or ultrasound scan showing no evidence of secondary dysmenorrhea, pain greater than or equal to 3 out of 10 on a numeric rating scale during the first three days of menses for at least two of the past three menstrual cycles, regular menstrual cycles (28 +/- 5 days) for the last three months, spoken and written English skills and able to give informed consent.

Exclusion criteria included a previous diagnosis of endometriosis or secondary dysmenorrhea, abdominal surgery in the previous three months, injectable or implant contraceptives (Depo Provera, Jadelle, Mirena) within the last three months, oral contraceptive usage started less than three months prior to enrolment, chronic pain conditions (>14 days per month with pain), current untreated mental health illness, neuropathic pain secondary to surgery, sterilisation.

### Recruitment

**Fig 1:**
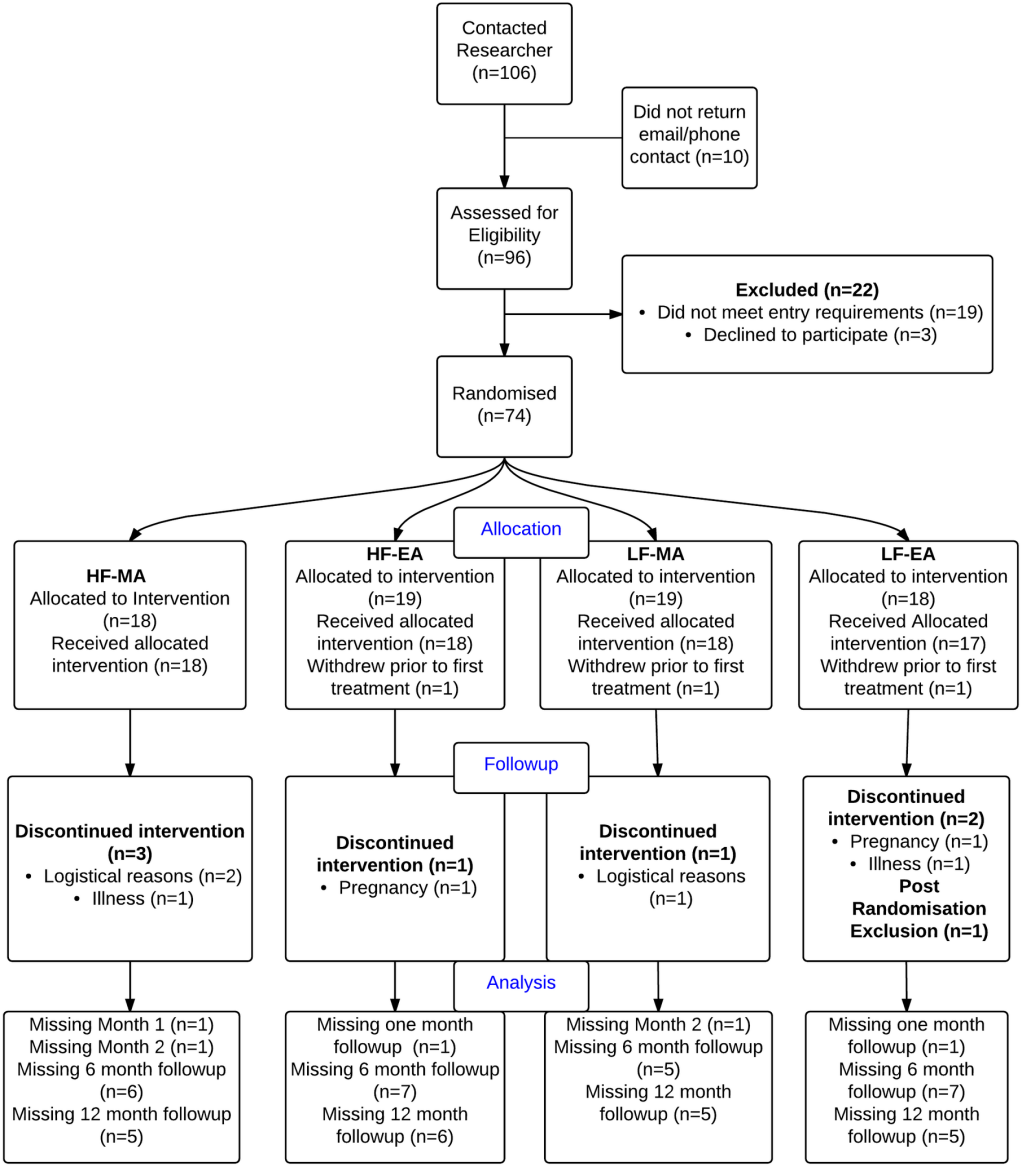
CONSORT flow diagram.

Based on prevalence data we expected the majority of potential participants to be aged under 25 [6]. Most of these women would not have discussed their condition with their general practitioner [10, 34] therefore we used similar promotion and recruitment strategies to Griffin [35] with promotion of the study focussed on distributing study information using university email lists, flyers and posters placed on campus. In addition, a Facebook advertisement and Google Ad-words were created in conjunction with the University of Western Sydney (UWS) social media committee. Both of these linked the participant with the UWS study website which provided more information and the contact details of the researcher. Facebook advertisements were targeted to women, aged 18–45 years living within 60 miles of Auckland and Wellington, New Zealand. Sixty miles was chosen as a suitable radius as it was considered unlikely that women outside this distance would be happy to travel in for treatment if randomised to either of the high-frequency groups. Upon contacting the primary investigator, women were assessed for initial eligibility. Women were then instructed to fill in a menstrual pain diary during their next menstrual period and this was assessed to confirm the presence of cycle length, pain levels and pain characteristics indicative of primary dysmenorrhea. Menstrual pain diary and baseline data were assessed to confirm eligibility before randomisation occurred. Fig 1 outlines the participant flow in the trial.

Randomisation was computer generated and allocation was concealed by way of an internet-based randomisation service, Sealed Envelope (www.sealedenvelope.com). Randomisation into one of four study groups using a 1:1:1:1 ratio was undertaken using the web interface by the primary investigator (MA). The four groups were, high frequency, manual acupuncture (HF–MA), high frequency, electro acupuncture (HF–EA), low frequency, manual acupuncture (LF–MA), low frequency, electro acupuncture (LF–EA).

### Treatment protocol

All women in the study were scheduled to receive 12 treatments over the course of three menstrual cycles. Women in the high frequency (HF) group received three treatments in the seven days prior to the estimated day one of the menstrual cycle. Women in the low frequency (LF) group received three treatments in the time between menses, approximately every seven to ten days’ dependent on cycle length. All groups received a treatment in the first two days of menses (day one or day two of the menses). In the manual acupuncture groups (-MA) all needles were stimulated by hand using tonifying, reducing or even method, based on the practitioner’s clinical judgement approximately 10–15 minutes after needle insertion was completed. In the electro-acupuncture groups (-EA) Two distal points (from the previously chosen point prescription) were selected by the practitioner and a 2Hz / 100Hz square wave pulse of 200ms duration was applied between each point for 20 minutes using an ITO ES-160 electro-acupuncture machine (Ito Co. Ltd, Japan). Intensity level was based on patient feedback; noticeable sensation present, but below the threshold for discomfort. All initial consultations were allocated 60 minutes, with follow-up consultations of 45 minutes’ duration.

The manualised protocol was based on data collected from an international survey of experienced women’s health practitioners [21] and focus groups [12]. Treatment was based on TCM’s eight principles and Zang Fu diagnosis. Once a TCM diagnosis had been ascertained, the practitioners had the flexibility with their point selection to address the diagnosed pattern of disharmony as per the treatment handbook. Each subsequent treatment session would allow for a confirmation or update on the TCM diagnosis and therefore point selection could vary from treatment to treatment. Multiple patterns of disharmony are common in dysmenorrhea[36] and this trial supported up to two concurrent patterns of disharmony, designated as root (primary) and secondary (branch) patterns. In all cases no more than seven acupuncture points were to be used. S1 Fig outlines how point selection occurred in both patients with one or two TCM patterns. Acupuncture points were needled bilaterally, except those where only one side was used clinically, such as opening extraordinary vessels such as the Chong Mai. Point location and needling depth was as specified in A Manual of Acupuncture [37]. Single use, stainless steel needles of varying gauge (.20 x 30mm or .25 x 40mm, DongBang, Korea) were used. *DeQi* (the arrival of Qi) is the sensation generated by the insertion and/or manipulation of an acupuncture needle in an acupuncture point [38]. In all groups DeQi was obtained for each acupuncture point at the beginning of the session and needles were retained for 20–30 minutes. Each pattern also has moxibustion as a compulsory, optional or forbidden component of the treatment. Indirect moxa was administered via smokeless moxa stick for 5–10 minutes on one of the selected acupuncture points. Each patient was given a diet and lifestyle advice sheet during their first treatment session. These were grounded in TCM theory.

### Blinding

Participants were not blinded to group allocation. Data entry was undertaken blind to the study group, and analysis was undertaken blind to group allocation.

### Outcomes

Changes in menstrual pain and other secondary symptoms were measured using a menstrual pain diary (MPD) that was designed by the authors (See S2 File). This was used to assess the changes in pain, analgesic intake, menstrual characteristics and secondary symptoms, and allowed women to self-rate the severity of their dysmenorrhea in both intensity and duration of pain, as well as the presence of secondary symptoms. The primary data endpoint used an 11-point numerical rating scale (NRS) for both “peak” and “average” pain each day, with 0 being no pain and 10 being the worst pain imaginable. The NRS has a high compliance rate, is easy to use, preferred by chronic pain patients and fits the compact format of a menstrual diary [39]. The diary also collected information on duration of menstrual pain each day (hours or “all day”), secondary menstrual symptoms, including breast tenderness, emotional changes, nausea etc. and analgesic usage (brand / pharmaceutical name, dosage and number of doses per day) This MPD could be filled in either in hard copy or using a webform. Both were identical in content and layout. The diary was completed by the participant at the conclusion of the day during either the menstrual period itself or one to two days prior to the menstrual period if they were experiencing sharp, stabbing, cramping or aching pain in the lower abdomen or lower back that they would normally associate with their period. The MPD was administered at baseline, at each of the three menstrual periods during the study, the menstrual period following the completion of the study, and at one, six and 12 months after the conclusion of the study.

Health related quality of life (HRQoL) was measured using the **36**-Item Short Form Health Survey (SF-36v2). The SF-36 has been shown to be valid and reliable [40], and has previously shown suitability for use in dysmenorrhea[41]. The SF-36v2 was self-administered at baseline (prior to the first acupuncture session) and again at one-month follow-up (post-trial completion).

An exit questionnaire was also used to determine self-reported improvement score, treatment satisfaction and to record which, if any, symptoms of primary dysmenorrhea improved (See S3 File).

The decision to calculate a responder rate was made *a posteriori*. The responder rate provides clinically meaningful information on the magnitude of pain reduction, in addition to the continuous outcome data. Responder rates were calculated for the primary outcome measures between baseline and one-month follow up, due to the significant loss to follow-up at 6 and 12 months. A recent review of chronic pain has shown that the threshold of a 30% reduction in pain scores is where patients feel a clinically important change has occurred [42]. However, the choice of responder rate can appear arbitrary and possibly bias the results, therefore providing a range of responder rates allows a more transparent view of the data [43]. Numeric data for responder rates is provided for 30%, 50% and 70% reductions from baseline.

### Sample size

This trial was designed explore a difference in peak pain intensity on the first 3 days of the menstrual period between groups as measured by the Numeric Rating Scale (NRS). A minimal clinically important 20% difference between groups was expected and based on previous study data [44], a standard deviation of 1.3 units on a pain scale was used. An alpha of 0.05 and power of 90% gives a total sample size of 60 women. A power calculation was performed using G*Power 3(G*Power: Statistical Power Analyses for Windows and Mac, Universität Düsseldorf). Previous studies [44, 45] had relatively low loss to follow-up (<15%), therefore a sample size of 72 women (18 per group) was used to account for the predicted loss to follow-up of 10–15%.

### Analysis

Data analysis used descriptive statistics and inferential statistics to examine the demographic and baseline characteristics. All statistical calculations were performed using IBM SPSS Statistics v23 (IBM Corporation). Age, BMI, age of menarche, analgesic usage, expectation, smoking and alcohol status, peak pain on the first three days of the menstrual period, average pain and SF-36 scores were compared between groups at baseline using one-way ANOVA for continuous data or chi-square for categorical data to ensure randomisation was successful. Differences at baseline are reported as means and standard deviations or frequencies and percentages. Both an ‘intention to treat’ approach and a per protocol analysis were undertaken for the primary and secondary endpoints. The menstrual symptoms score was calculated as the mean number of symptoms per participant per day. Average pain for each menstrual cycle was calculated as the mean of each days ‘average pain’ score over each menstrual period. Pre-planned contrasts were to compare mode of stimulation (MA vs EA) and frequency of treatment (HF vs LF) and the effect of these combinations via the four different groups. Differences in the primary and secondary outcome measures were analysed using linear mixed model analysis of variance with either i) group and time as fixed effects or ii) mode of stimulation, frequency of treatment and time as fixed effects. In all linear mixed models subject was a used as a random effect. Any interactions between group and time, mode of stimulation and time and frequency of treatment and time were investigated. Due to the small sample size all p-values are exploratory. All pre-planned *post hoc* tests used Sidak for multiple pairwise comparisons. In the secondary analysis where means have been adjusted by SF-36 Role Physical, expectation of benefit and current alcohol consumption, all relevant tables report both adjusted and unadjusted means.

## Results

A total of 106 women expressed interest in the study (See Fig 1). Seventy-four women met the eligibility criteria and were randomised to one of the four groups. Sixty-three (85%) women completed the treatment phase of the trial. Ten participants withdrew during the treatment phase of the trial. Withdrawal from the trial was equally distributed across groups, with none of the women citing any group-related concerns as reason for withdrawal.

The demographic and menstrual characteristics of the women at baseline are shown in Table 1. One-way ANOVA and Pearson Chi-square showed three areas of imbalance between the groups at baseline, in SF-36 role physical (F(3,69) = 1.12, p = 0.008), in expectation of benefit (χ(6) = 13.205, p = 0.04) and in current alcohol consumption (χ(3) = 8.562, p = 0.036). These were used as co-variates in the secondary analysis.

**Table 1:** Baseline demographic and health characteristics of women at trial entry.

	HF-MA (n = 18)	HF-EA (n = 19)	LF-MA (n = 19)	LF-EA (n = 18)	Total (N = 74)	p-value
	mean (SD)	mean (SD)	mean (SD)	mean (SD)	mean (SD)	
*Demographic*						
**Age**	29.9 (7.2)	31.2 (7.4)	31.1 (6.6)	29.3 (5.6)	30.4 (6.7)	0.80
BMI (n %)*						
Underweight	0 (0.0)	2 (10.5)	1 (5.2)	0 (0.0)	3 (4.0)	
Normal	9 (50.0)	8 (42.1)	13 (68.4)	11 (61.1)	41 (55.4)	0.50
Overweight	4 (22.2)	6 (31.5)	4 (21.0)	4 (22.2)	18 (24.3)	
Obese	5 (27.7)	3 (15.7)	1 (5.2)	3 (16.6)	12 (16.2)	
**Previously given birth**						
No	14 (77.7)	16 (84.2)	13 (68.4)	15 (83.3)	58 (78.4)	0.62
Yes	4 (22.2)	3 (15.7)	6 (31.6)	3 (16.6)	16 (21.6)	
*Menstrual characteristics*						
**Age of Menarche (years)**	12.5 (1.7)	12.7 (1.4)	13.2 (1.6)	12.3 (0.9)	12.7 (1.5)	0.31
**Age of onset of dysmenorrhea (years)**	15.8 (6.8)	13.7 (1.5)	15.9 (5.8)	13.3 (1.5)	14.7 (4.7)	0.19
**Length of menstrual cycle (days)**	28.1 (1.8)	28.7 (2.1)	27.8 (2.0)	28.6 (2.1)	28.3 (2.0)	0.50
**Length of menses (days)**	5.3 (1.9)	4.9 (0.8)	5.4 (1.2)	5.6 (1.7)	5.3 (1.2)	0.49
**Additional Menstrual Symptoms**						
No	0 (0)	0 (0)	0 (0)	0 (0)	0 (0)	
Yes	18 (100)	19 (100)	19 (100)	18 (100)	74 (100)	1.0
**Pain relief with analgesia**						
No—Pain still present	2 (11.1)	2 (10.5)	0 (0.0)	0 (0.0)	4 (5.4)	
Yes—Partial Relief	13 (72.2)	16 (84.2)	15 (78.9)	17 (94.4)	61 (82.4)	0.37
Yes—Complete relief	3 (16.6)	1 (5.2)	3 (15.7)	1 (5.6)	8 (10.8)	
**Currently drinking alcohol**						
No	2 (11.1)	3(15.7)	9(47.3)	3(16.6)	17 (22.9)	0.03*
Yes	16 (88.9)	16(84.2)	10 (52.6)	15(83.3)	57 (77.0)	
**Expectation of benefit from acupuncture**						
Unsure	9 (50.0)	4 (21.0)	10 (52.6)	7 (38.8)	30 (40.5)	
Probably will help	4 (22.2)	14 (73.6)	8 (42.1)	8 (44.4)	34 (46.0)	0.040*
Definitely will help	5 (27.8)	1 (5.2)	1 (5.2)	3 (16.6)	10 (13.5)	
**SF-36 HRQoL**						
Physical function	55.8 (2.9)	55.0 (3.9)	52.8 (8.4)	53.7 (7.4)	54.3 (6.1)	0.46
Role physical	54.8 (5.0)	46.4 (9.7)	50.2 (7.8)	47.6 (8.0)	49.7 (8.4)	0.01 *
Bodily pain	49.1 (7.4)	43.6 (5.3)	44.8 (9.3)	43.4 (7.4)	44.9 (7.7)	0.08
General health	51.3 (10.6)	50.4 (10.4)	52.8 (9.9)	51.6 (7.7)	51.5 (9.6)	0.90
Vitality	48.1 (9.8)	45.4 (7.8)	48.2 (9.9)	48.9 (6.5)	47.7 (8.6)	0.61
Social function	50.7 (8.2)	43.9 (11.3)	44.9 (10.7)	47.6 (7.0)	46.7 (9.7)	0.15
Role emotional	49.6 (6.6)	47.6 (8.9)	47.6 (8.9)	49.0 (8.0)	48.4 (8.1)	0.83
Mental health	51.0 (6.3)	50.3 (5.8)	47.3 (8.1)	49.6 (7.2)	49.5 (6.9)	0.38
Overall mental component	48.1 (8.1)	44.8 (10.0)	46.1 (9.4)	47.9 (8.7)	46.7 (9.0)	0.68
Overall physical component	54.1 (6.6)	49.4 (6.7)	51.4 (6.3)	49.3 (6.9)	51.0 (6.8)	0.13

Continuous data presented as mean (SD). Categorical data as N (%).

* between group comparison significant (P<0.05)

However, not all women received all 12 treatments, mostly due to logistical issues around timing of treatments with regards to the start of menses. Twenty-five participants (35.2%) received the full 12 study treatments. The mean number of treatments was 10.6 [95% CI 10.22 to 10.99] for all women who had at least one study treatment. There was no difference in the number of treatments given between groups (F(3,59) = 1.855, p = 0.147).

Table 2 outlines peak pain, average pain, duration of pain and analgesic usage for each of the groups at each time point for adjusted and unadjusted ITT analysis. See S4 File for results of the per-protocol analysis.

**Table 2:** Changes in menstrual pain and analgesic usage between baseline and follow up.

Group	HF–MA n = 18		HF–EA n = 19		LF–MA n = 19		LF–EA n = 18	
	Unadjusted Mean [95% CI]	Adjusted Mean [95% CI]	Unadjusted Mean [95% CI]	Adjusted Mean [95% CI]	Unadjusted Mean [95% CI]	Adjusted Mean [95% CI	Unadjusted Mean [95% CI]	Adjusted Mean [95% CI]
**Peak pain (Day 1–3 of menstrual period)**								
Baseline	4.5 [3.5 to 5.4]	4.4 [3.4 to 5.5]	5.1 [4.2 to 6.0]	5.7 [4.7 to 6.8]	5.3 [4.4 to 6.2]	5.5 [4.5 to 6.5]	4.7 [3.7 to 5.7]	5.0 [3.9 to 6.0]
Month 1	3.4 [2.5 to 4.4]	3.4 [2.4 to 4.4]	4.0 [3.1 to 5.0]	4.6 [3.6 to 5.7]	4.7 [3.8 to 5.7]	4.9 [4.0 to 5.9]	3.8 [2.8 to 4.8]	4.0 [3.0 to 5.0]
Month 2	3.7 [2.8 to 4.6]	3.7 [2.6 to 4.7]	4.0 [3.0 to 4.9]	4.6 [3.5 to 5.6]	4.1 [3.2 to 5.1]	4.3 [3.3 to 5.3]	3.9 [2.9 to 4.9]	4.1 [3.1 to 5.2]
Month 3	3.4 [2.5 to 4.4]	3.4 [2.3 to 4.5]	3.3 [2.3 to 4.2]	3.9 [	3.0 [2.1 to 4.0]	3.2 [2.2 to 4.2]	3.8 [2.8 to 4.8]	4.1 [3.0 to 5.1]
1 month follow up	2.7 [1.8 to 3.6]	2.7 [1.6 to 3.8]	3.7 [2.8 to 4.7]	4.3 [3.2 to 5.4]	3.4 [2.5 to 4.4]	3.7 [2.7 to 4.6]	3.7 [2.8 to 4.8]	4.0 [2.9 to 5.0]
6 month follow up	2.8 [1.9 to 3.8]	2.8 [1.8 to 3.9]	3.6 [2.6 to 4.5]	4.2 [3.1 to 5.2]	3.5 [2.5 to 4.5]	3.7 [2.7 to 4.7]	3.7 [2.8 to 4.7]	4.0 [2.9 to 5.0]
12 month follow up	2.9 [1.9 to 3.9]	2.9 [1.8 to 4.0]	3.6 [2.6 to 4.5]	4.2 [3.1 to 5.2]	3.8 [2.8 to 4.7]	4.0 [3.0 to 4.9]	4.0 [3.0 to 4.9]	4.2 [3.2 to 5.3]
**Average Pain**								
Baseline	2.4 [1.7 to 3.1]	2.6 [1.8 to 3.3]	2.7 [2.1 to 3.4]	3.3 [2.6 to 4.1]	3.2 [2.5 to 3.9]	3.5 [2.8 to 4.2]	2.6 [1.9 to 3.3]	2.9 [2.2 to 3.6]
Month 1	2.2 [1.5 to 2.9]	2.3 [1.6 to 3.1]	2.6 [1.9 to 3.3]	3.3 [2.5 to 4.0]	2.7 [2.1 to 3.4]	3.0 [2.4 to 3.7]	2.4 [1.7 to 3.1]	2.7 [2.0 to 3.4]
Month 2	2.5 [1.8 to 3.3]	2.7 [2.0 to 3.5]	2.4 [1.7 to 3.1]	3.0 [2.3 to 3.8]	2.5 [1.8 to 3.1]	2.8 [2.1 to 3.5]	2.2 [1.5 to 2.9]	2.5 [1.8 to 3.2]
Month 3	2.1 [1.4 to 2.8]	2.3 [1.5 to 3.0]	2.0 [1.4 to 2.8]	2.7 [1.9 to 3.4]	1.7 [1.0 to 2.4]	2.0 [1.7 to 3.1]	2.2 [1.5 to 3.0]	2.5 [1.8 to 3.2]
1 month follow up	1.6 [1.0 to 2.3]	1.8 [1.0 to 2.6]	2.3 [1.6 to 3.0]	2.9 [2.2 to 3.7]	2.0 [1.4 to 2.8]	2.4 [1.7 to 3.1]	2.2 [1.5 to 3.0]	2.6 [1.8 to 3.3]
6 month follow up	1.6 [0.9 to 2.3]	1.7 [1.0 to 2.5]	2.3 [1.6 to 3.0]	2.9 [2.1 to 3.6]	2.0 [1.4 to 2.8]	2.3 [1.6 to 3.0]	2.2 [1.5 to 3.0]	2.5 [1.8 to 3.2]
12 month follow up	1.8 [1.1 to 2.5]	2.0 [1.2 to 2.7]	2.3 [1.6 to 3.0]	2.9 [2.1 to 3.6]	2.3 [1.6 to 3.0]	2.6 [1.9 to 3.3]	2.4 [1.6 to 3.0]	2.7 [1.9 to 3.4]
**Duration of pain (hours per day)**								
Baseline	4.0 [1.9 to 6.2]	3.0 [0.9 to 5.2]	6.6 [4.5 to 8.8]	7.4 [5.3 to 9.6]	7.0 [4.8 to 9.1]	7.0 [5.0 to 9.0]	6.5 [4.3 to 8.7]	6.5 [4.4 to 8.6]
Month 1	4.0 [1.7 to 6.1]	2.9 [0.8 to 5.1]	6.4 [4.3 to 8.6]	7.2 [5.1 to 9.4]	7.3 [5.2 to 9.5]	7.4 [5.4 to 9.4]	6.5 [4.4 to 8.8]	6.6 [4.5 to 8.8]
Month 2	3.0 [0.9 to 5.2]	2.1 [0.0 to 4.2]	6.4 [4.2 to 8.5]	7.2 [5.0 to 9.4]	5.0 [2.8 to 7.1]	5.0 [3.0 to 7.0]	5.3 [3.2 to 7.6]	5.4 [3.3 to 7.5]
Month 3	2.9 [0.8 to 5.1]	2.0 [0.0 to 4.1]	4.0 [1.9 to 6.1]	4.8 [2.7 to 7.0]	4.2 [3.0 to 6.3]	4.2 [2.2 to 6.3]	4.6 [2.4 to 6.8]	4.6 [2.5 to 6.7]
1 month follow up	3.1 [0.9 to 5.3]	2.1 [0.0 to 4.3]	5.2 [3.1 to 7.4]	6.0 [3.9 to 8.2]	5.2 [3.0 to 7.3]	5.2 [3.2 to 7.2]	4.0 [1.8 to 6.2]	4.1 2.0 to 6.2]
6 month follow up	3.0 [0.8 to 5.2]	2.0 [0.0 to 4.1]	5.1 [3.0 to 7.2]	6.0 [3.8 to 8.1]	5.3 [3.1 to 7.4]	5.3 [3.3 to 7.3]	4.0 [1.8 to 6.2]	4.0 [2.0 to 6.1]
12 month follow up	3.3 [1.1 to 5.5]	2.3 [0.1 to 4.5]	4.9 [2.8 to 7.1]	5.8 [3.6 to 8.0]	5.5 [3.4 to 7.7]	5.6 [3.6 to 7.6]	4.3 [2.1 to 6.5]	4.3 [2.2 to 6.5]
**Analgesic medication (doses per day)**								
Baseline	0.52 [0.32 to 0.74]	0.34 [0.16 to 0.52]	0.7 [0.49 to 0.9]	0.55 [0.37 to 0.72]	0.47 [0.26 to 0.67]	0.38 [0.21 to 0.56]	0.51 [0.30 to 0.72]	0.43 [0.25 to 0.61]
Month 1	0.25 [0.05 to 0.47]	0.25 [0.07 to 0.43]	0.62 [0.41 to 0.82]	0.60 [0.43 to 0.78]	0.33 [0.13 to 0.54]	0.30 [0.12 to 0.48]	0.43 [0.21 to 0.64]	0.42 [0.24 to 0.60]
Month 2	0.36 [0.15 to 0.56]	0.37 [0.19 to 0.54]	0.51 [0.30 to 0.71]	0.43 [0.25 to 0.61]	0.29 [0.09 to 0.49]	0.29 [0.11 to 0.47]	0.43 [0.21 to 0.64]	0.42 [0.24 to 0.60]
Month 3	0.34 [0.13 to 0.55]	0.28 [0.10 to 0.46]	0.46 [0.26 to 0.67]	0.40 [0.23 to 0.58]	0.25 [0.05 to 0.46]	0.21 [0.04 to 0.39]	0.38 [0.17 to 0.60]	0.39 [0.20 to 0.57]
1 month follow up	0.28 [0.07 to 0.48]	0.22 [0.05 to 0.40]	0.58 [0.37 to 0.78]	0.51 [0.33 to 0.70]	0.27 [0.07 to 0.47]	0.26 [0.09 to 0.44]	0.44 [0.23 to 0.65]	0.43 [0.25 to 0.61]
6 month follow up	0.36 [0.16 to 0.58]	0.28 [0.10 to 0.46]	0.55 [0.34 to 0.75]	0.50 [0.32 to 0.67]	0.27 [0.07 to 0.47]	0.26 [0.09 to 0.44]	0.47 [0.26 to 0.68]	0.45 [0.26 to 0.63]
12 month follow up	0.48 [0.27 to 0.70]	0.36 [0.17 to 0.53]	0.54 0[.33 to 0.75]	0.49 [0.31 to 0.67]	0.31 [0.10 to 0.51]	0.29 [0.1 to 0.47]	0.58 [0.37 to 0.80]	0.52 [0.34 to 0.71]

### Primary outcome

#### Abdominal pain

For the primary outcome of peak pain during the first three days of menses at the 12 month follow-up, all groups showed a significant reduction in peak abdominal pain over time, (*F*(6, 1519) = 12.3, p < .0001) and neither mode of stimulation (MA vs EA) (p = 0.48) nor frequency of treatment (LF vs HF) (p = 0.362) showed a significant effect. There was no significant difference between groups (p = 0.597) or any group*time interaction (p = 0.506). A secondary analysis which included baseline co-variates as fixed effects did not significantly alter any of the outcomes.

### Secondary outcomes

#### Abdominal pain

For the secondary outcome of average abdominal pain over the menstrual period all groups showed reductions in average abdominal pain over time (*F*(6, 2324) = 7.98, p < .0001) and neither mode of stimulation (MA vs EA) (p = 0.61) nor frequency of treatment (LF vs HF) (p = 0.74) showed a significant effect. There was no difference between the four groups (p = 0.846) or any group*time interaction (p = 0.105). A secondary analysis with baseline covariates did not significantly alter any of the outcomes.

#### Duration of menstrual pain

For the duration of menstrual pain each day all groups showed reductions in the duration of menstrual pain over time, (*F*(6, 2317) = 12.721, p < .0001) and neither mode of stimulation (MA vs EA) (p = 0.61) nor frequency of treatment (LF vs HF) (p = 0.74) showed a significant effect. There was no significant difference between groups (p = 0.247) or any group*time interaction (p = 0.287). A secondary analysis with baseline covariates did show a difference between groups (*F*(3, 2313) = 3.151, p = 0.024). A post-hoc pairwise analysis showed that the HF-MA group had significantly shorter duration of pain at baseline compared to HF-EA (MD -4.4, 95%CI -7.9 to -1.50, p = 0.003) and LF-MA (MD -3.9, 95%CI -6.9 to 1.0, p = 0.008), at month 1 compared to HF-EA (MD -4.4, 95%CI -7.3 to -1.4, p = 0.004), LF-MA (MD -4.4, 95%CI -7.4 to -1.5, p = 0.003), and LF-EA (MD -3.7, 95%CI -6.6 to -0.8, p = 0.01) and at month 2 compared to HF-EA (MD -5.1, 95%CI -8.1 to -2.2, p = 0.001). This difference was no longer significant from month 3 onwards.

#### Analgesic medication

Medication usage was the mean number of doses per day of menses of analgesic medication. Analgesic medication usage reduced in all groups over time, (*F*(6, 1519) = 3.4, p = 0.002) and mode of stimulation (MA vs EA) showed a significant effect, (*F*(1, 2343) = 5.3, p = 0.02) while frequency of treatment (LF vs HF) did not (p = 0.338). Post-hoc pairwise analysis showed the manual acupuncture groups had lower analgesic intake at month 1 (MD -0.230, 95% CI -0.437 to -0.023, p = 0.03) and 1 month follow-up (MD -0.234, 95% CI MD -0.441 to -0.027, p = 0.027), 6 month follow-up (MD -0.228, 95% CI MD -0.40 to -0.051, p = 0.012) and 12 month follow-up (MD -0.182, 95% CI MD -0.361 to -0.004, p = 0.045). There was no significant difference between groups (p = 0.151) or any group*time interaction (p = 0.921). Secondary analysis which included baseline co-variates as fixed effects did not significantly alter any of the outcomes.

#### Secondary symptoms

**Fig 2:**
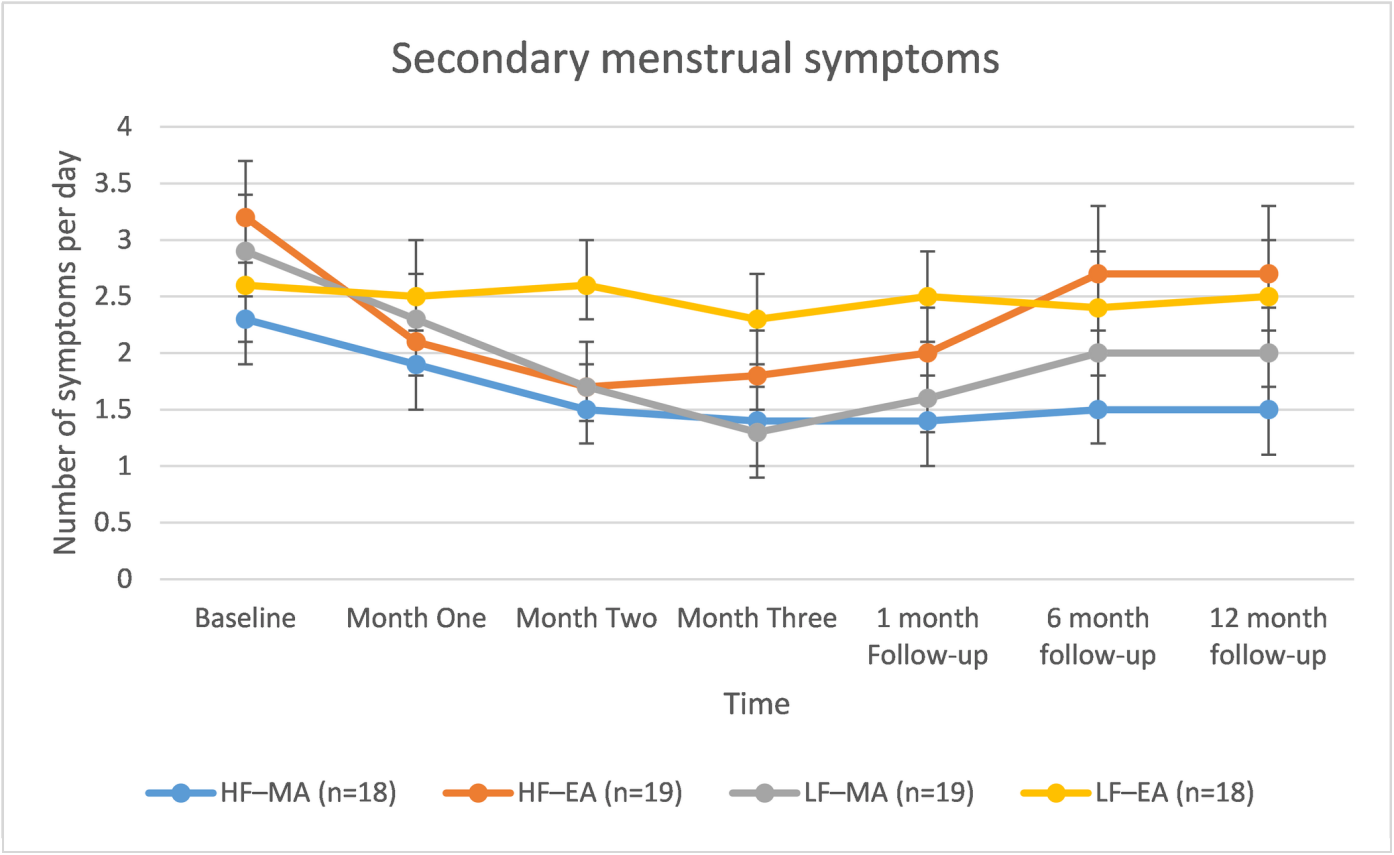
Secondary menstrual symptoms by group over time. Error bars are 95% confidence intervals based on estimated margin means.

There was a decrease in menstrual symptoms over time (*F*(6, 2313) = 8.4, p<0.001) which was different between groups (*F*(18, 2313) = 2.49, p<0.001), see Fig 2. Mode of stimulation (MA vs EA) showed a significant difference in the number of menstrual symptoms at different time points, (*F*(1, 2320) = 4.2, p<0.001) while frequency of treatment (LF vs HF) did not (p = 0.858). *Post-hoc* pairwise analysis showed that the LF–EA group had significantly higher menstrual symptom count at Month Two to all groups, at Month Three compared with HF–MA (MD -0.975, 95% CI -1.7 to -0.2, p = 0.005) and LF–MA (MD -0.908, 95% CI -0.14 to -1.6, p = 0.011) and at one-month follow-up to HF–MA (MD -1.1, 95% CI -0.32 to -1.9, p = 0.001) and LF–MA (MD -0.92,95% CI -0.12 to -1.7, p = 0.013). At six and 12-month follow-up the HF-MA group had significantly lower secondary symptom count compared to both HF-EA (MD -1.12, 95% CI -1.9 to -0.30, p = 0.003) and LF-EA (MD -0.956, 95% CI -1.79 to -0.12, p = 0.018).

#### Responder rate

The proportion of responders with clinically significant pain reduction of 30% in their peak pain was highest in the HF–MA group across all three days (55%, 60% and 61% of women respectively) and over a third of women in this group had a 70% reduction in their peak pain (See Table 3). Almost three-quarters (72%) of the women in the HF–MA group had a clinically significant 30% reduction in their average pain, with 69% of the LF–MA group, 61% of the LF–EA group and the 47% of the HF–EA group achieving this reduction. Almost 60% of the LF–MA group and 55% of the HF–MA group had a 50% reduction in average pain from baseline to one-month follow-up. The electro-acupuncture groups had lower scores, with 42% and 33% of the HF–EA and LF–EA groups achieving a 50% reduction.

**Table 3:** Proportion of responders at each pain-reduction percentage between baseline and follow-up.

		Day One			Day Two			Day Three	
	*30%*	*50%*	*70%*	*30%*	*50%*	*70%*	*30%*	*50%*	*70%*
**HF–MA**	55.6%	50.0%	38.9%	61.1%	44.4%	38.9%	61.1%	50.0%	38.9%
**HF–EA**	36.8%	26.3%	21.1%	42.1%	31.6%	26.3%	47.4%	47.4%	42.1%
**LF–MA**	52.6%	26.3%	15.8%	42.1%	31.6%	15.8%	52.6%	47.4%	36.8%
**LF–EA**	50.0%	44.4%	27.8%	38.9%	27.8%	22.2%	16.7%	11.1%	5.6%

#### Health-related quality of life (SF-36v2)

Overall, both the high-frequency groups showed a greater number of improvements in the SF-36 domains (six domains) compared to the low-frequency groups (two domains). Health related quality of life measured by the SF-36 improved across a number of domains, between baseline and one-month follow-up (see Table 4). Paired t-tests showed that there were improvements in the domains of role physical (p = 0.0029), bodily pain (p = 0.0002), vitality (p = 0.028), social function (p = 0.0067) and overall physical component (p = 0.001) between baseline and one-month follow-up. A one-way ANOVA showed no difference between groups at the one month follow-up time point.

**Table 4:** SF-36 scores at one-month follow-up.

	HF–MA (n = 18)	HF–EA (n = 19)	LF–MA (n = 17)	LF–EA (n = 15)	Total (n = 69)	
	mean (SD)	mean (SD)	mean (SD)	mean (SD)	mean (SD)	p-value (baseline to follow-up)
Physical function	55.6 (2.6)	56.2 (1.2)	53.3 (5.1)	55.6 (3.5)	55.2 (3.5)	0.207
Role physical	54.2 (5.0)	50.7 (6.7)*	52.1 (4.4)	49.9 (6.8)	51.8 (5.9)	0.0029*
Bodily pain	50.7 (6.4)	48.7 (9.0)*	48.0 (7.4)*	47.2 (6.7)	48.7 (7.4)	0.0002*
General health	52.8 (11.2)	51.7 (9.1)	52.99 (7.9)	53.4 (7.9)	53.0 (9.0)	0.12
Vitality	52.7 (8.3)*	47.6 (8.9)	49.8 (10.8)	47.4 (9.5)	49.5 (9.4)	0.028*
Social function	53.9 (7.0)*	48.6 (10.0)*	47.3 (9.2)	46.6 (6.7)	49.2 (8.7)	0.0067*
Role emotional	50.7 (7.2)	47.9 (7.8)	48.6 (9.9)	47.5 (8.4)	48.75 (8.4)	0.209
Mental health	51.3 (7.6)	51.2 (7.2)	48.8 (10.7)	49.2 (8.6)	50.2 (8.5)	0.267
Overall mental component	50.6 (9.6)*	47.3 (9.5)	47.1 (12.2)	46.0 (9.3)	47.89 (10.1)	0.142
Overall physical component	54.5 (5.8)	53.1 (5.2)*	52.8 (6.7)	52.9 (6.8)*	53.3 (6.0)	0.001*

* indicates significant change between baseline and one-month follow-up.

#### Self-rated symptom improvement

At the end of the intervention the mean self-rated improvement score by group was HF–MA 7.4 (95% CI 6.13 to 8.67), HF–EA 6.75 (95% CI 5.15 to 8.35), LF–MA 7.47 (95% CI 6.44 to 8.50), and LF–EA 6.5 (95% CI 5.07 to 7.93). Overall self-rated improvement score, independent of group was 7.05 (95% CI 6.43 to 7.67). A one-way ANOVA showed no difference between groups in self-rated improvement (F(3,58) = .577, p = 0.632). Participants were asked to list the symptoms, if any, that they thought improved the most during the trial. Women reported that abdominal cramps were the most common symptom to improve, with 36 women indicating abdominal pain or cramping showed significant improvement. Emotional changes (13 women) and back and leg pain (15 women) were also commonly reported improvements.

#### Safety

Fifty-two adverse events (AE) occurred over 702 acupuncture sessions, giving an overall adverse event rate of 7.4% (95% CI 5.6 to 9.6) (see Table 5). There was no difference in AE rate between groups. Most of the events were minor and self-limiting. Bruising (haematoma) was the most common adverse event (50% of all AE or 3.7% of treatments) with post treatment soreness occurring in 1.4% of treatments and fatigue in 1.1% of treatments.

**Table 5:** Adverse events during the treatment period.

Adverse event	% of total treatments (95% CI)
Bruising	3.7% [2.5 to 5.3]
Post-treatment soreness	1.4% [0.74 to 2.6]
Fatigue	1.1% [0.54 to 2.2]
Feeling faint	0.28% [0.1 to 1.1]
Itching	0.14% [0.1 to 0.89]
Bowel changes	0.14% [0.1 to 0.89]
Burns	0.14% [0.1 to 0.89]

## Discussion

Acupuncture treatment, regardless of group, using a manualised acupuncture protocol, showed significant clinical improvements in all menstrual pain scores recorded; ‘peak pain’ over the first three days of the menstrual period and ‘average pain’ over the entire menstrual period, and that reduction was sustained for twelve months. This was supported by a similar significant reduction across all other measures of menstrual impact; duration of pain, analgesic usage, secondary menstrual symptoms and a high self-rated improvement score. Electro-acupuncture did not deliver greater pain reduction than manual acupuncture. Manual acupuncture groups tended to have a higher responder rate, significantly greater reduction in secondary symptoms, and lower analgesic usage. With respect to health related quality of life six domains improved from baseline in the HF groups, with only two improving from baseline in the LF groups. There was no difference between manual and electro-acupuncture in terms of domains improved.

The lack of superiority of electro-acupuncture in terms of pain reduction was unexpected. Electro-acupuncture in the trial was delivered at a 2/100Hz frequency, which has been shown to provide maximal opioid gene expression by combining both high and low frequencies to provide maximal pain relief[46]. This high frequency–low frequency stimulation pattern is very common in gynaecological disorders[47], and is identical to the frequency used in many other trials of electro-acupuncture for primary dysmenorrhea[48–51]. Animal models suggest that needling at REN4, SP8 and SP6, used in 80%, 55% and 98% of all study treatments respectively, regulates neuro-endocrine activities, including levels of progesterone[52]; and needling SP6 has been shown to increase ovarian blood flow via a reflex response in rats[53]. A similar reflex response exists in humans, increasing uterine blood flow by reducing sympathetic activity on vasoconstrictor fibres that innervate the uterus[25, 54]. Uterine blood flow is often reduced in women with primary dysmenorrhea[55, 56] and increasing uterine blood flow appears to be related to some of the analgesic benefits of acupuncture in primary dysmenorrhea[50, 57]. The magnitude of the impact of needling SP6 and, to a lesser extent, REN4 and SP8, may be larger than stimulation-specific differences. Another plausible possibility is that the time course of increased endogenous opioid release caused by electro-acupuncture is too short to contribute to a noticeable overall reduction in menstrual pain [58].

In addition to reductions in pain, many women had a reduction in their secondary menstrual symptoms. Inflammation is a primary cause of primary dysmenorrhea [59] and a possible cause of PMS symptoms, including mood changes, bloating and breast tenderness [60]. The wide range of improvements in both pain and PMS related symptoms, especially mood changes, seen in this trial provide support for acupuncture’s posited anti-inflammatory effects[24], future research including suitable bio-makers would provide more information on these possible pathways.

Analgesic medication usage was lower amongst the manual acupuncture groups. As analgesic usage was allowed *ad libitum* women may have used analgesic medication to achieve an acceptable level of pain. Therefore, the lower usage of analgesic medication amongst these two groups suggests that without analgesic usage there may have been more pronounced differences between these two groups. The reasons for this lower use are unclear, as electro-acupuncture should provide at least the same amount of relief as manual acupuncture, as the needles were stimulated to obtain DeQi prior to electro-stimulation, thereby delivering a manual acupuncture-like stimulus prior to the addition of the electro-acupuncture pulse[61]. The changes in the HRQoL SF-36 domains suggest that after the course of acupuncture treatment, participants had less problems with work or daily activities as a result of their physical health, less pain and subsequent limitations due to pain, more energy, and less interference from physical and emotional problems in social activities[62], all of which are commonly experienced by women with primary dysmenorrhea [8, 63].

In terms of pain reduction, the trial findings were similar to results provided by previous studies. Direct comparison to studies performed in China[29, 48–51] with very short timescales of measurement post-treatment are problematic as these may not represent meaningful changes over the entire menses. Comparisons with other trials outside of China tend to show similar results to this study. Absolute pain reductions were less than Witt (Germany)[45] and Helms (US) [64] but similar to that found by Smith (Australia)[44]. Comparison with Helms [64] is complicated by the fact that the sample size was very small, showing no between group differences and the use of a custom pain scale that was not validated. The greater absolute pain reduction found by Witt [45] may be due to the nature of the intervention, where physicians were allowed to choose treatment without restriction, or due to the greater sample size, almost ten times the size of this study. However, the responder rates (>30% reduction in average pain from baseline) were similar to Witt’s, showing that the percentage pain reduction from baseline was relatively equal between the two studies. Similarly to Smith [44] and Witt [45], reductions in pain in this study appeared to be sustained or increased after the trial period ended.

There were no between group differences in all pain outcome measures, despite large differences in responder rates between groups. This is most likely due to the exploratory nature of the study, which was not powered to detect small between group differences. A *post-hoc* power analysis shows that a sample size of 192 would be required to determine a 20% difference between groups for average pain. While a difference of >30% from baseline is considered clinically important, the size of differences between groups that are important to participants appears to be dependent on the condition treated[43]. In this case, these between group differences do not appear to significantly alter how successful women felt their treatment was, supported by the fact there was no difference in self-reported improvement score between groups, however, future studies with significantly more participants would be required to answer this question.

The strengths of this exploratory study were that it recruited a group of participants who were representative of women in the community with dysmenorrhea, having a variety of secondary symptoms, including back pain, fatigue, breast tenderness, nausea, headaches and bowel changes, consistent with many other populations with primary dysmenorrhea[8, 9, 34]. Analgesic usage amongst the study participants was very prevalent (81%), almost identical to the prevalence of analgesic usage in Australian women with dysmenorrhea (80%)[65]. There are several limitations on this exploratory study: Due to the high loss to follow-up at 6 and 12 months the significant use of last observation carried forward in a small sample such as this could contribute to over-estimating the duration of the treatment effect in the intention to treat analysis, however a per-protocol analysis without using last observation carried forward showed similar results. The RCT, due to its design in comparing effectiveness of differing doses of acupuncture, did not have a usual care group. The changes observed over the 12 month period are unlikely to be due to natural reductions in the pain from primary dysmenorrhea[66]. Witt (Germany) [45] showed a 15% reduction in average menstrual pain in the usual care group at 3 months while our study found a 24% reduction in average pain independent of group allocation at the same time point. This suggests an additional benefit of acupuncture but our ability to determine the extent to which acupuncture treatment is responsible for the changes in pain, menstrual symptoms and health-related quality of life observed in the study is limited.

## Conclusion

This exploratory study suggests acupuncture administered over three menstrual cycles gave both statistically and clinically significant reductions in menstrual pain compared to baseline and persisted for 12 months, however there was no significant difference in pain intensity between groups. Electro-acupuncture was well tolerated but did not always provide the same magnitude of improvements as manual acupuncture in reducing secondary menstrual symptoms and analgesic medication intake. Manual acupuncture provided the same or greater pain relief as electro-acupuncture, but with less analgesic medication required to achieve this pain reduction. Treatment timing appears to play a small role, with high frequency of treatment providing greater improvements in health-related quality of life. This exploratory trial suggests that there may be an effect of both treatment timing and mode of stimulation, but possibly only of small clinical benefit. Future adequately powered studies are needed to confirm the role of both mode of stimulation and treatment timing and to provide a more accurate indication of the clinical impact of altering these dose components on menstrual pain.

## Supporting information

S1 Fig Diagnosis and point selection. (DOCX)

S1 File Manualised acupuncture trial protocol. (PDF)

S2 File Menstrual pain diary. (PDF)

S3 File Trial exit questionnaire. (PDF)

S4 File Per protocol analysis. (DOCX)

S1 Consort Checklist (DOC)

S1 Appendix Protocol for the RCT. (DOCX)

S2 Appendix Data analysis file ITT (SPSS). (SAV)

S3 Appendix Data analysis file PP (SPSS). (SAV)

S4 Appendix Model fit data. (XLSX)

S5 Appendix Raw SF-36 data. (XLSX)

S6 Appendix Demographic dataset. (SAV)
