# Supplementary figures and images for: The role of treatment timing and mode of stimulation in the treatment of primary dysmenorrhea with acupuncture: An exploratory randomised controlled trial
Source: PLoS One. 2017 Jul 12;12(7):e0180177. doi: 10.1371/journal.pone.0180177 (PMC5507497; doi:10.1371/journal.pone.0180177)

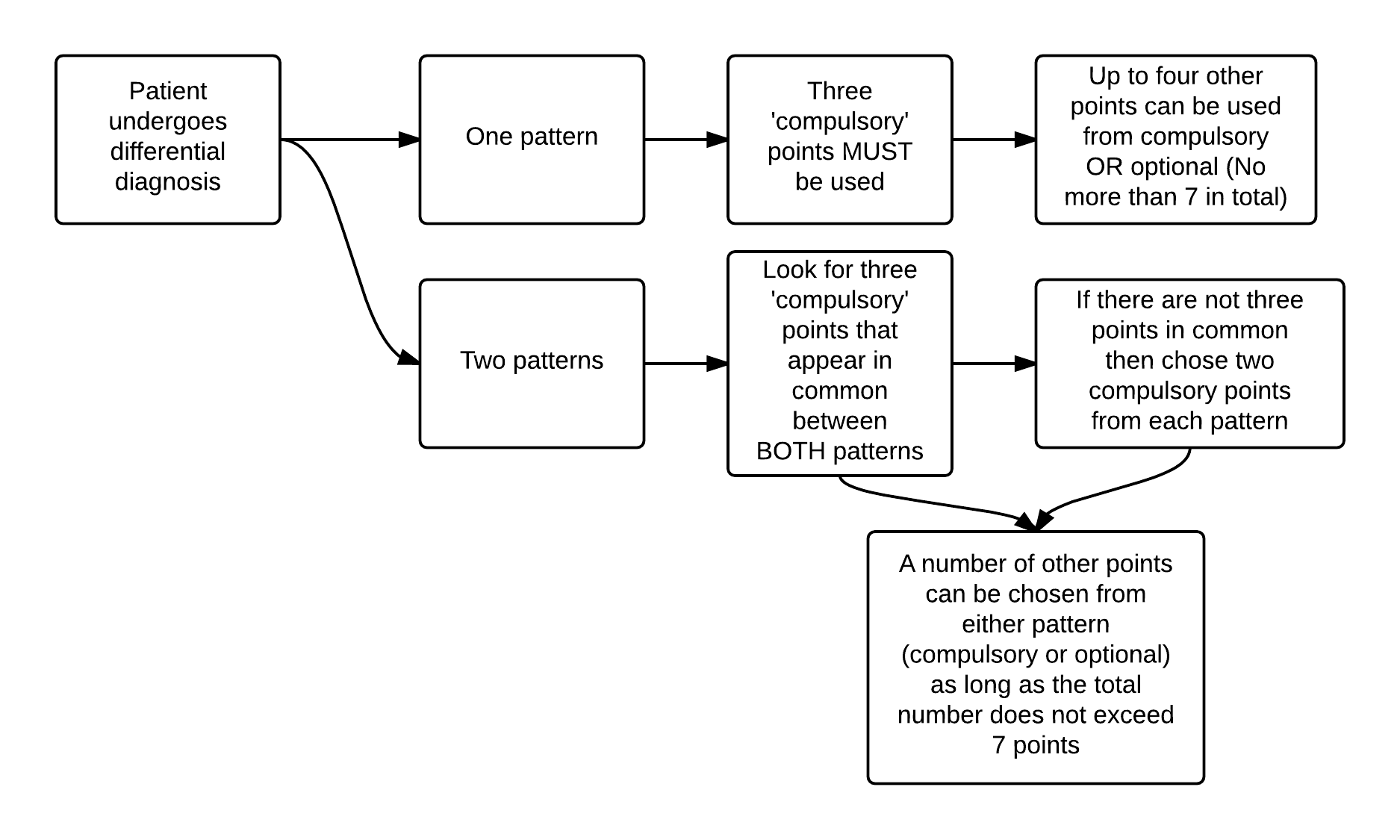

Supplement: S1 Fig — (DOCX) [file pone.0180177.s001.docx]
