# Supplementary material for: The role of treatment timing and mode of stimulation in the treatment of primary dysmenorrhea with acupuncture: An exploratory randomised controlled trial
Source: PLoS One. 2017 Jul 12;12(7):e0180177. doi: 10.1371/journal.pone.0180177 (PMC5507497; doi:10.1371/journal.pone.0180177)
Supplement: S1 File — (PDF) [file pone.0180177.s002.pdf]

Protocol Manual for Study Acupuncturists in the Acupuncture for Treatment of Dysmenorrhea study

This trial is based around using a manualised version of standard acupuncture treatments which has been designed in consultation with experienced practitioners through interviews and focus groups.

This manualisation allows considerable flexibility, in order that the treatment can match patient variability. The protocol is not too broad, such that “anything goes” as this would mean that it would be more difficult to attribute any impact of treatment to the different interventions we are using, frequency and stimulation of treatment. The protocol is also not too narrow, as we want the treatments to broadly reflect how acupuncturists normally work in clinical practice.

The important features in this protocol are:

1. Take the case history, including current orthodox medical diagnosis if present, and undertake traditional acupuncture diagnostic assessments. This needs to be entered on the Case Report Forms (CRF) which have been provided.

2. Identify your patient’s patterns of disharmony using the provided list of patterns of disharmony that have been developed. Multiple patterns are common in clinical practice and therefore can be used in this trial. You will be asked to justify your clinical reasoning for each pattern.

3. Implement your treatment strategy based on your traditional acupuncture diagnosis, to be applied flexibly at each treatment on an individual patient basis.

4. In devising the treatment strategy you will use the set of practitioner contributed points for each pattern as well as other cointerventions such as moxabustion if indicated.

5. The point selection is to be adapted as necessary at subsequent sessions, drawing on relevant theoretical considerations.

6. Select acupuncture needles of an appropriate length and gauge, based on relevant theoretical considerations.

7. Administer acupuncture needles to an appropriate depth, obtaining de qi where required, based on relevant theoretical considerations.

8. Use appropriate needling techniques as discussed in the protocol, relevant to the group allocation.

9. Retain needles for an appropriate period of time, based on this protocol.

10. Integrate relevant prescribed self-help activities at appropriate times based on the protocol.

11. Integrate into the treatment strategy relevant life-style support with the rationale being based on Chinese medicine theory identified above.

12. Use relevant explanations to provide for your patient any necessary information about their condition, their traditional acupuncture diagnosis, the aims and methods of the acupuncture treatment, and their prognosis.

13. Devise with your patient a treatment plan that covers the number and frequency of treatments, based on their group allocation and their availability.

14. Make judgements about the possibility of secondary dysmenorrhea if symptoms suggest it, and where appropriate, encourage patients to seek help from their GP.

Initial Contact and Consultation:

Prospective participants in the study will be screened by the primary investigator and once enrolled will be given your contact details and will be asked to make contact with you directly. Once the participants have enrolled you will be contacted with their name, study ID and group allocation. If you do not receive this prior to the patient making an appointment please contact the primary investigator ASAP. Do not guess the patients group allocation!

For the duration of the treatment phase of the research you will deal directly with the patient when arranging times, making bookings etc. There is an EZY-Book service set up to help you with this. If you have issues with non-compliance such as missing appointments then please advise the primary investigator.

Apart from differences in treatment frequency and mode of stimulation which are prescribed by the different groups all patients should receive the same components of a normal acupuncture consultation (ie history taking, pulse and tongue diagnosis etc). Its very important that participants receive the same level of care as they would in private practice.

Please allow 60 minutes for the first consultation and 45-60 minutes for the followup consultations depending on how your clinic arranges booking times.

Study participants will have already filled in a baseline menstrual diary before coming to see you. They do not need to bring anything to their consultation with them for the purposes of this study.

BEFORE *the first treatment please ask them to fill in an SF-36 form. Add their study ID and mark 'Baseline' on this form. Please return this to Mike Armour when next sending off documents package. This should be done before the first treatment session begins.*

Please use the form '*Case Report Form - Initial Consultation*' to guide your questioning within your normal history taking framework and complete as many sections as possible. It is important that you consult the sheets on the following pages as a guide on your final differential diagnosis. Please keep any completed Case Report Form in a secure,locked filing cabinet.

Differential Diagnosis and Point selection:

Once you have completed your history taking please use the Pattern and Point guide in your practitioner pack to guide your differential diagnosis. There is space on the case report form where we ask you to discuss your clinical reasoning, please be concise but accurate in what you felt were the key indicators for this pattern.

Multiple patterns are common in clinic and this trial supports up to two patterns. Please designate one as the primary(or root) and one as the secondary (or branch) pattern if possible.

- If you have ONE pattern then you need to choose three points from the 'Vital' category under your pattern of disharmony. In addition if it is needed in your clinical judgement then you may use -up to- another four points from either the vital or optional category (Please do not use more than seven points).

- If you have TWO patterns of disharmony then if possible choose three points that are common to both patterns. If this is not possible then choose two points from each patterns 'Vital' category. In addition if it is needed in your clinical judgement you can use -up to- another three points from the vital or optional catagory from EITHER pattern (Please do not use than more than seven points)

- If you wish to use Back Shu and front points then please split the treatment into two equal halves.

NB Master/Couple points to open Extraordinary Vessels (ie SP4 + PC6) count as one point for the purposes of maximum number of points.

Needling and co-interventions:

All point locations are as per Peter Deadman's '*A Manual of Acupuncture'.* Please swab the relevant locations using single use alcohol swabs prior to insertion. Needle length and gauge are at your clinical discretion and should be based on both the anatomical location and the body shape of the participant.

Insert needles using clean needle technique as per NZRA guidelines.

Please achieve DeQi in **all** groups after insertion.

- In groups assigned to have manual acupuncture, once DeQi is obtained please manipulate the needles **once** more during the session with stimulation (tonify, reduce or even) based on your clinical judgement. Needles should be retained for approximately 20 minutes with a maximum of 30 minutes, based on your clinical judgement.

- In groups assigned to Electro-acupuncture allow a short time for initial DeQi to subside or reduce after needle insertion then please attach electrodes between two points of your choosing. Distal points such as Stomach 36, Spleen 6, Spleen 8 and Liver 3 are recommended over the use of abdominal points for participant comfort. An alternating waveform of 2/100Hz needs to be used with stimulation level at a level which is both noticeable and comfortable for the participant. If at any stage the participant becomes uncomfortable or distressed please reduce the intensity of stimulation. Electro-acupuncture stimulation should be in place for approximately 20 minutes unless the participant shows distress or discomfort.

The use of moxibustion has been listed as either Required or Optional under each pattern of disharmony. If it is listed as Required and you do not use it, please explain your reasoning in the Case Report Form.

When using moxibustion please apply it indirectly (via moxa stick) on up to 3 acupuncture points of your choosing for 10 minutes or until the points are warm, whichever is first. Moxibustion is to be done after needle insertion. Do NOT use direct moxibustion.

During the **FIRST** session please discuss and then give to the participant an appropriate handout based on their pattern of disharmony (primary or root pattern only). These are included in your practitioner pack. Discuss this advice in the last 5-10 minutes of the treatment session. Try to give small, manageable pieces of advice each session, as per the handout, rather than overwhelming the patient.

Concluding the session:

At the end of the session please ensure all needles are removed. If there is any significant bleeding or bruising please enter on the Case Report Form. Advise the participant that they may feel slightly fatigued and ensure they do not drive until feeling alert.

Make sure the participant has been given, if relevant, a copy of the lifestyle/diet advice to take away. Ensure they have a current menstrual diary.

Please book patients in for future sessions. Remember that each patient is treated for three menstrual cycles.

If participants are in the high frequency group you will need to book them in to see you a total of 3 times in the seven days prior to the start of menses and once in the first two days of menses.

If participants are in the low frequency group you need to book them in to see you once every seven days for a total of three times per month before menses and once in the first two days of menses.

If there are scheduling issues due to unresolvable scheduling conflict for the participant please try and achieve as close to the target as possible and advise the primary investigator via email.

Subsequent sessions:

Please use the *Case Report Form - Second consultation onwards*. Always enquire about any adverse events (see Adverse Events Sheet in your practitioner pack) and note them on the Case Report Form. Any uncommon or unexpected events should be reported to the Primary Investigator as soon as possible via email or phone.

Returning documentation:

Once a participant has finished their three menstrual cycles please provide them with an exit questionnaire and pre-stamped envelope and ask them to fill in and return via post.

After **every** menstrual cycle please send the completed Case Report Forms and Menstrual diary for the previous cycle to the Primary Investigator.

Ethical Research Practice:

It is very important that as a research acupuncturist in this study you follow the guidelines on Good Clinical Practice (CPMP/ICH/135/95)

- Please do not treat any conditions other than Primary Dysmennorhea (ie colds/flu).
- Please ensure all details on the Case Report Forms are correct and a truthful record of the treatment.
- Please ensure that you provide the treatment as designated by this protocol and the participants group allocation. Do not substitute other points.
- Please do not prescribe herbal remedies or dietary supplements to the participant during the treatment period.
- It is a breach of ethics to see the participant for other conditions during the treatment period.
- You will be observed at random treating a trial participant at least once during the study. You will be given advance notice of this.
- Participation is voluntary and participants should never experience any duress to stay in the study.
- Participants names should never be written on the Case Report Forms. Ensure that only the Study ID is used.
- All trial documentation must be keep in either a secure, locked filing cabinet or password secured electronic file.

Any queries or concerns you can contact the Primary Investigator as any time

Principal Investigator:

Mike Armour
Phone: 098326307
Mobile: 021906644
Email: m.armour@uws.edu.au

Postal Address:
1 Kay Road
Swanson
Auckland 0612
