# Supplementary material for: The role of treatment timing and mode of stimulation in the treatment of primary dysmenorrhea with acupuncture: An exploratory randomised controlled trial
Source: PLoS One. 2017 Jul 12;12(7):e0180177. doi: 10.1371/journal.pone.0180177 (PMC5507497; doi:10.1371/journal.pone.0180177)
Supplement: S2 File — (PDF) [file pone.0180177.s003.pdf]

Acupuncture for Period Pain:
Menstrual Diary

COMPLEMED

Centre for Complementary Medicine Research

University of Western Sydney

Bringing knowledge to life

Thank you for taking part in our study on Acupuncture for period pain. To assess the effect of treatment we need you to fill in this menstrual diary. It's simple and easy to fill in, just read the following instructions and take a look at the example below. Please return this using the prepaid envelope provided once your period has finished.

Important:
Please start the diary when either: Your period begins, even if there is no pain on the first day **or** You have lower abdominal/back pain even if its one or two days prior to the period beginning. We need a *complete* diary of the period so please do not start after Day 1.

Please fill the diary until your period finishes. So if you have a day of pain prior to your period and then five days of bleeding you will need to fill this in for six days.

Please remember, fill this diary in on any day where you have lower back pain or cramping,sharp, stabbing or aching lower abdominal pain, even if your period hasn't started.

Each page has the following columns on it, this is what each column is used for (please read before filling in)

1) **Date** means the current date, so if you are making an entry on the 21st of June, enter 21/6 or 21 June.
2) **Day of cycle** is the number of days since the beginning of your last period. If your period has started then the first day is day 1. If you are starting the diary the day before your period begins then if it's been 27 days since the beginning of your last period this would be day 27.
3) **Menstrual flow**. This records the 'flow' of your period. If you are not bleeding then leave blank. Generally, small clots are less than the size of your little finger nail while large clots are bigger than that.
4) **Pain**. This is measured on a scale of 0 through 10, from no pain (0) to the worst pain imaginable (10). This should be pain related to the period itself (ie pain in your abdomen or lower back). Other pain, not in this area, (such as headaches that come with your period)

should be noted in the "Other Symptoms" section, please do not enter this pain level here. **Worst Pain** is the peak or highest pain level for that day. **Average Pain** is the average pain over the whole day. **Average Pain** should not be higher than **Worst Pain.** If you know when your pain started and finished please enter this as well. You can also write ‘Whole Day’ if its always there.

5) Other symptoms. These are symptoms which are related to period pain in addition to the abdominal pain (or ‘cramps’). Tick all that apply.
6) Medications: Please note any pain relief you use along with the dosage. So if you used 2 ibuprofen 200mg tablets then enter 2 x Ibuprofen 200mg here. Please make an note for each dose you take.
7) Other: Here you can enter any other remedies you use to help deal with your pain (ie hot water bottle). You can also use this to make a note if you are off work or school because of your period pain. There is space to enter any other information you think is important.

To help here is an example of the first day of the period filled in

Date	Day of Cycle	Menstrual Flow	Pain	Other Symptoms	Medication	Other
13 Feb	1	☐ Spotting<br>☒ Light<br>☐ Medium<br>☐ Heavy<br><br>☒ Small Clots<br>☐ Large Clots<br>☐ No Clots	Worst 6 / 10<br><br>Average 4 /10<br><br>Pain Start 8am<br><br>Pain Ends 3pm<br><br>7 hours of pain today	☐ Breast tenderness<br>☐ Fluid Retention<br>☒ Back pain<br>☐ Leg/Thigh pain<br>☒ Bloating<br>☐ Emotional changes<br>☒ Nausea<br>☐ Vomiting<br>☐ Headache<br>☐ Dizziness<br>☐ Bowel changes	2 x ibuprofen 200mg<br><br>2 x ibuprofen 200mg<br><br>__ x __________<br><br>__ x __________	☒ Hot Water Bottle<br>☐ Rest<br>☒ Other<br>Went to bed early<br><br>☐ Absent from Work or School due to period pain

Date	Day of Cycle	Menstrual Flow	Pain	Other Symptoms	Medication	Other
		☐ Spotting<br>☐ Light<br>☐ Medium<br>☐ Heavy<br><br>☐ Small Clots<br>☐ Large Clots<br>☐ No Clots	Worst ____ / 10<br><br>Average ___ /10<br><br>Pain Start ____<br><br>Pain Ends ____<br><br>____ hours of pain today	☐ Breast tenderness<br>☐ Fluid Retention<br>☐ Back pain<br>☐ Leg/Thigh pain<br>☐ Bloating<br>☐ Emotional changes<br>☐ Nausea<br>☐ Vomiting<br>☐ Headache<br>☐ Dizziness<br>☐ Bowel changes	__ x __________<br><br>__ x __________<br><br>__ x __________<br><br>__ x __________	☐ Hot Water Bottle<br>☐ Rest<br>☐ Other<br>________________<br><br>________________<br><br>☐ Absent from Work or School due to period pain
		☐ Spotting<br>☐ Light<br>☐ Medium<br>☐ Heavy<br><br>☐ Small Clots<br>☐ Large Clots<br>☐ No Clots	Worst ____ / 10<br><br>Average ___ /10<br><br>Pain Start ____<br><br>Pain Ends ____<br><br>____ hours of pain today	☐ Breast tenderness<br>☐ Fluid Retention<br>☐ Back pain<br>☐ Leg/Thigh pain<br>☐ Bloating<br>☐ Emotional changes<br>☐ Nausea<br>☐ Vomiting<br>☐ Headache<br>☐ Dizziness<br>☐ Bowel changes	__ x __________<br><br>__ x __________<br><br>__ x __________<br><br>__ x __________	☐ Hot Water Bottle<br>☐ Rest<br>☐ Other<br>________________<br><br>________________<br><br>☐ Absent from Work or School due to period pain

Date	Day of Cycle	Menstrual Flow	Pain	Other Symptoms	Medication	Other
		☐ Spotting<br>☐ Light<br>☐ Medium<br>☐ Heavy<br><br>☐ Small Clots<br>☐ Large Clots<br>☐ No Clots	Worst ____ / 10<br><br>Average ___ /10<br><br>Pain Start ____<br><br>Pain Ends ____<br><br>____ hours of pain today	☐ Breast tenderness<br>☐ Fluid Retention<br>☐ Back pain<br>☐ Leg/Thigh pain<br>☐ Bloating<br>☐ Emotional changes<br>☐ Nausea<br>☐ Vomiting<br>☐ Headache<br>☐ Dizziness<br>☐ Bowel changes	__ x __________<br><br>__ x __________<br><br>__ x __________<br><br>__ x __________	☐ Hot Water Bottle<br>☐ Rest<br>☐ Other<br>________________<br><br>________________<br><br>☐ Absent from Work or School due to period pain
		☐ Spotting<br>☐ Light<br>☐ Medium<br>☐ Heavy<br><br>☐ Small Clots<br>☐ Large Clots<br>☐ No Clots	Worst ____ / 10<br><br>Average ___ /10<br><br>Pain Start ____<br><br>Pain Ends ____<br><br>____ hours of pain today	☐ Breast tenderness<br>☐ Fluid Retention<br>☐ Back pain<br>☐ Leg/Thigh pain<br>☐ Bloating<br>☐ Emotional changes<br>☐ Nausea<br>☐ Vomiting<br>☐ Headache<br>☐ Dizziness<br>☐ Bowel changes	__ x __________<br><br>__ x __________<br><br>__ x __________<br><br>__ x __________	☐ Hot Water Bottle<br>☐ Rest<br>☐ Other<br>________________<br><br>________________<br><br>☐ Absent from Work or School due to period pain

Date	Day of Cycle	Menstrual Flow	Pain	Other Symptoms	Medication	Other
		☐ Spotting<br>☐ Light<br>☐ Medium<br>☐ Heavy<br><br>☐ Small Clots<br>☐ Large Clots<br>☐ No Clots	Worst ____ / 10<br><br>Average ___ /10<br><br>Pain Start ____<br><br>Pain Ends ____<br><br>____ hours of pain today	☐ Breast tenderness<br>☐ Fluid Retention<br>☐ Back pain<br>☐ Leg/Thigh pain<br>☐ Bloating<br>☐ Emotional changes<br>☐ Nausea<br>☐ Vomiting<br>☐ Headache<br>☐ Dizziness<br>☐ Bowel changes	__ x __________<br><br>__ x __________<br><br>__ x __________<br><br>__ x __________	☐ Hot Water Bottle<br>☐ Rest<br>☐ Other<br>________________<br><br>________________<br><br>☐ Absent from Work or School due to period pain
		☐ Spotting<br>☐ Light<br>☐ Medium<br>☐ Heavy<br><br>☐ Small Clots<br>☐ Large Clots<br>☐ No Clots	Worst ____ / 10<br><br>Average ___ /10<br><br>Pain Start ____<br><br>Pain Ends ____<br><br>____ hours of pain today	☐ Breast tenderness<br>☐ Fluid Retention<br>☐ Back pain<br>☐ Leg/Thigh pain<br>☐ Bloating<br>☐ Emotional changes<br>☐ Nausea<br>☐ Vomiting<br>☐ Headache<br>☐ Dizziness<br>☐ Bowel changes	__ x __________<br><br>__ x __________<br><br>__ x __________<br><br>__ x __________	☐ Hot Water Bottle<br>☐ Rest<br>☐ Other<br>________________<br><br>________________<br><br>☐ Absent from Work or School due to period pain

Office use only	Date	Signed
LOG		
CHECK		
DATA Entry		
