# Supplementary material for: The role of treatment timing and mode of stimulation in the treatment of primary dysmenorrhea with acupuncture: An exploratory randomised controlled trial
Source: PLoS One. 2017 Jul 12;12(7):e0180177. doi: 10.1371/journal.pone.0180177 (PMC5507497; doi:10.1371/journal.pone.0180177)
Supplement: S3 File — (PDF) [file pone.0180177.s004.pdf]

University of Western Sydney
Bringing knowledge to life

Office use only	Date	Signed
LOG		
CHECK		
DATA Entry		

Acupuncture for the treatment of Primary Dysmenorrhea

Thank you for participating in our study, since you have now completed your final treatment we would like you to answer this short questionnaire. This will help us evaluate our trial and informs future trial design.

The following questions are about your experiences over the course of the trial. Please tick all the boxes that apply

1. What did you enjoy or think was beneficial about being in the trial?

- I did not enjoy being in the trial ☐
- I enjoyed the extra attention to my health and wellbeing ☐
- I enjoyed my relationship with the acupuncturist ☐
- I enjoyed being able to lie down and have 'time out' ☐
- I found the lifestyle/self help advice my acupuncturist gave me helpful ☐
- I enjoyed an alternative way of looking at health and wellbeing ☐
- Being able to help others with period pain via research ☐
- Other (please explain)____

____

2. **What did you not enjoy or think was detrimental about being in the trial ?**

- There was nothing I did not enjoy ☐
- I spent more time thinking about my period pain ☐
- I did not like having to fill in the menstrual diary ☐
- I found the acupuncture unpleasant or uncomfortable ☐
- I found the appointments took up too much time or hard to schedule ☐
- I did not enjoy my relationship with my acupuncturist ☐
- My symptoms got worse after being in the trial ☐
- Other (please explain)____

____

3. **If we were doing another, similar study on using acupuncture for period pain would you participate or would you recommend it to friends or family who had period pain ?**

I would definitely participate/recommend it	☐
I would probably participate/recommend it	☐
I don't know	☐
I would probably not participate/recommend it	☐
I would definitely not participate/recommend it	☐

4. **How effective did you find the acupuncture treatment for your period pain ?**

Please rate the change (or lack of) that you had in your period related symptoms at the end of the three months of treatment. This includes lower abdominal pain ("cramps"), lower back pain, and any other symptoms you noted in your diary such as headaches or emotional changes. 0 is no change at all and 10 is significant improvements.

___ / 10

5. **If you did have improvement in your symptoms during the trial, what symptom(s) improved the most?**

This can be any period related symptoms, not just restricted to pain. For example if your pre-period sugar cravings reduced you can tell us about it here. You can list as many here as you like or if you got no change in your symptoms please just write 'None'

____

____

____

____

6. **What were the three most important features of the sessions with your acupuncturist ?**

This can be anything you enjoyed, from having a chance to lie down and relax to finding it helpful to have someone listen to you. If you feel that there was nothing important about these sessions please note it here. This is more about your experience in the trial than the results you got. Please list up to THREE features here

1. ______________________________________

2. ______________________________________

3. ______________________________________

6. **Why did you find these important ?**

7. **Do you have any other comments you'd like to make ?**

Thank you for filling in this questionnaire, we appreciate your time!

Date: Study ID:
