# Supplementary material for: The role of treatment timing and mode of stimulation in the treatment of primary dysmenorrhea with acupuncture: An exploratory randomised controlled trial
Source: PLoS One. 2017 Jul 12;12(7):e0180177. doi: 10.1371/journal.pone.0180177 (PMC5507497; doi:10.1371/journal.pone.0180177)
Supplement: S4 File — (DOCX) [file pone.0180177.s005.docx]

|  | **HF-MA** | **HF-EA** | **LF-MA** | **LF-EA** |  |
| --- | --- | --- | --- | --- | --- |
|  | Unadjusted Mean [95% CI] | Unadjusted Mean [95% CI] | Unadjusted Mean [95% CI] | Unadjusted Mean [95% CI] | *P* value |
| **Peak pain (Day 1-3 of menstrual period)** |  |  |  |  | 0.566 |
| Baseline | 4.7 [3.7 to 5.8] | 5.1 [4.1 to 6.0] | 5.4 [4.5 to 6.4] | 4.6 [3.7 to 5.7] |  |
| Month 1 | 3.3 [2.3 to 4.4] | 3.8 [2.9 to 4.9] | 4.8 [3.8 to 5.8] | 3.6 [2.6 to 4.6] |  |
| Month 2 | 3.6 [2.5 to 4.6] | 3.7 [2.8 to 4.7] | 3.9 [2.9 to 4.9] | 3.7 [2.7 to 4.6] |  |
| Month 3 | 3.2 [2.2 to 4.3] | 3.0 [2.0 4.0] | 2.8 [1.9 to 3.9] | 3.6 [2.6 to 4.6] |  |
| 1 month follow up | 2.4 [1.3 to 3.4] | 3.5 [2.5 to 4.5] | 3.3 [2.4 to 4.4] | 3.5 [2.5 to 4.5] |  |
| 6 month follow up | 2.9 [1.0 to 4.7] | 2.5 [0.3 to 4.7] | 3.3 [1.7 to 5.0] | 3.8 [1.6 to 5.9] |  |
| 12 month follow up | 2.6 [1.3 to 3.9] | 2.8 [1.4 to 4.3] | 3.9 [2.6 to 5.2] | 4.7 [2.6 to 6.9] |  |
| **Average Pain** |  |  |  |  | 0.624 |
| Baseline | 2.5 [1.7 to 3.2] | 2.8 [2.1 to 3.5] | 3.3 [2.6 to 4.0] | 2.5 [1.8 to 3.3] |  |
| Month 1 | 2.1 [1.4 to 2.9] | 2.6 [1.9 to 3.3] | 2.8 [2.1 to 3.6] | 2.3 [1.6 to 3.0] |  |
| Month 2 | 2.4 [1.7 to 3.2] | 2.2 [1.5 to 2.9] | 2.4 [1.7 to 3.1] | 2.1 [1.4 to 2.8] |  |
| Month 3 | 1.9 [1.1 to 2.7] | 2.0 [1.3 to 2.7] | 1.7 [0.9 to 2.4] | 2.1 [1.4 to 2.8] |  |
| 1 month follow up | 1.3 [0.6 to 2.1] | 2.2 [1.6 to 2.9] | 2.0 [1.3 to 2.8] | 2.1 [1.4 to 2.9] |  |
| 6 month follow up | 1.0 [0 to 2.2] | 1.3 [0 to 2.8] | 1.7 [0.7 to 2.8] | 2.3 [0.8 to 3.8] |  |
| 12 month follow up | 1.5 [.7 to 2.4] | 1.9 [0.9 to 2.8] | 2.6 [1.7 to 3.5] | 2.9 [1.5 to 4.4] |  |
| **Duration of pain (hours per day)** |  |  |  |  | 0.282 |
| Baseline | 4.6 [2.2 to 6.0] | 6.9 [5.1 to 8.7] | 6.9 [5.1 to 8.7] | 5.6 [3.7 to 7.4] |  |
| Month 1 | 4.0 [2.1 to 5.9] | 6.4 [4.5 to 8.2] | 7.2 [5.4 to 9.0] | 5.4 [3.5 to 7.2] |  |
| Month 2 | 3.2 [1.3 to 5.1] | 6.0 [4.2 to 7.8] | 4.2 [2.4 to 6.0] | 4.1 [2.3 to 5.9] |  |
| Month 3 | 2.9 [1.6 to 4.8] | 3.6 [1.8 to 5.5] | 3.8 [2.0 to 5.6] | 3.3 [1.4 to 5.1] |  |
| 1 month follow up | 3.2 [1.3 to 5.1] | 5.0 [3.1 to 6.8] | 4.8 [3.0 to 6.7] | 2.8 [0.9 to 4.7] |  |
| 6 month follow up | 2.2 [0 to 5.2[ | 5.3 [1.6 to 9.0] | 4.2 [1.6 to 6.9] | 4.1 [0.4 to 7.8] |  |
| 12 month follow up | 3.2 [1.0 to 5.4] | 4.3 [1.8 to 6.8] | 5.0 [2.8 to 7.3] | 5.3 [1.8 to 8.9] |  |
| **Analgesic medication (doses per day)** |  |  |  |  | 0.030* |
| Baseline | 0.39 [0.20 to 0.58] | 0.60 [0.43 to 0.78] | 0.36 [0.18 to 0.54] | 0.40 [0.22 to 0.58] |  |
| Month 1 | 0.22 [0.03 to 0.41] | 0.59 [0.41 to 0.77] | 0.25 [0.07 to 0.43] | 0.41 [0.23 to 0.60] |  |
| Month 2 | 0.41 [0.22 to 0.60] | 0.38 [0.20 to 0.56] | 0.24 [0.06 to 0.42] | 0.41 [0.23 to 0.60] |  |
| Month 3 | 0.29 [0.10 to 0.47] | 0.36 [0.18 to 0.54] | 0.17 [0.0 to 0.35] | 0.38 [0.20 to 0.56] |  |
| 1 month follow up | 0.19 [0.0 to 0.38] | 0.49 [0.31 to 0.67] | 0.21 [0.03 to 0.39] | 0.43 [0.24 to 0.61] |  |
| 6 month follow up | 0.42 [0.10 to 0.73] | 0.24 [0.0 to 0.63] | 0.20 [0.0 to 0.48] | 0.75 [0.35 to 1.1] |  |
| 12 month follow up | 0.4 [0.18 to 0.62] | 0.66 [0.4 to 0.91] | 0.26 [0.03 to 0.48] | 1.0 [0.66 to 1.4] |  |
