# Supplementary material for: The role of treatment timing and mode of stimulation in the treatment of primary dysmenorrhea with acupuncture: An exploratory randomised controlled trial
Source: PLoS One. 2017 Jul 12;12(7):e0180177. doi: 10.1371/journal.pone.0180177 (PMC5507497; doi:10.1371/journal.pone.0180177)
Supplement: S1 Appendix — (DOCX) [file pone.0180177.s007.docx]

*Protocol*

**Acupuncture for Primary Dysmenorrhea: Examining the role of frequency of treatment and mode of stimulation on clinical outcomes**

**Protocol Number** Version 3

**Date** 20 June 2013

**Grant:** University of Western Sydney

**Principal Investigator:** Mike Armour, PhD candidate, Centre for Complementary Medicine Research, University of Western Sydney

**Supervisors:**

A/Prof Caroline Smith, Centre for Complementary Medicine Research, University of Western Sydney

Prof Hannah Dahlen, School of Midwifery and Nursing, University of Western Sydney

Dr Xiaoshu Zhu, School of Science and Health, , University of Western Sydney

Prof Cindy Farquhar, Department of Obstetrics and Gynecology, Faculty of Medical and Health Science, University of Auckland

**Study Centre:**

The Centre for Complementary Medicine Research, The University of Western Sydney, Locked Bag 1797, Penrith, NSW 2751

Table of Contents

1. Introduction 3

1.1 Background 3

1.2 Rationale for Study 5

2. Study Objectives 5

3. Study Design 6

4. Study Population 6

4.1 Sample Size 6

4.2 Recruitment 6

4.3 Inclusion Criteria 6

4.4 Exclusion Criteria 6

5. Participant selection and enrollment 6

5.1 Identifying participants 7

5.2 Screening for eligibility: 7

5.3 Consenting participants 7

5.4 Ineligible and non-recruited participants 7

5.5 Randomisation 7

6. Investigational therapy and controls 8

6.1 Acupuncture 8

6.2 Control group 10

6.3 Prior and Concomitant Medications 10

6.4.1 Permitted medications 10

7. Study Assessments 10

7.1 Safety Assessments 10

7.2 Study assessments 10

8. Data Collection 10

9. Statistics and Data Analysis 12

9.1 Sample Size calculation: 12

9.2 Proposed Analysis: 12

10. Monitoring and quality assurance 12

10.1 Project management and trial management group 12

11. Good Clinical Practice Module 13

11.1 Ethical conduct of the study 13

11.2 Investigator responsibilities 13

12. Reporting, Publication and Notification of Results 14

12.1 Authorship policy 14

12.2 Publication 14

13. References 14

# 1. Introduction

## 1.1 Background

Dysmenorrhea can be described as painful uterine cramps of menstrual origin (1). Dysmenorrhea can be classified as primary or secondary. Primary dysmenorrhea (PD) is pain in the absence of any organic cause and is most common in women under the age of 25, with pain starting within three years of menarche (2,3).Characteristic symptoms of primary dysmenorrhea are crampy, colicky spasms of pain in the suprapubic area occurring within 8-72 hours of menstruation and peaking within the first few days as menstrual flow increases (1,3,4). In addition to painful cramps many women with primary dysmenorrhea experience back and thigh pain, headaches, diarrhea, nausea and vomiting (1,3).

Between 43-91% of women under 20 (5) and 16.8 – 81% of women of reproductive age (6) experience dysmenorrhea and is a leading cause of regular absence from school or work amongst reproductive aged women (7) and reduced classroom or academic performance amongst Australian adolescents (8) and is thought to cause significant economic impact (9).

Common biomedical treatments are Non-Steroidal Anti Inflammatory drugs (NSAIDs) such as ibuprofen and the Combined Oral Contraceptive (COC) (2,10)These interventions are effective for many women however there are significant adverse events associated with both interventions (10,11) with regular monthly ingestion raising concerns about long-term risks in NSAIDs (12-14).

While NSAIDs and COC are effective for many women approximately 25% of women have pain that is refractory to either of these standard treatments (15,16)

*Acupuncture*

Complementary and Alternative medicine (CAM) is commonly used by women in Australia and New Zealand with chronic, unresolved health problems (17-21). Given the chronic nature of dysmenorrhea and the fact that many women are not receiving significant symptom resolution from standard therapies it is logical that many women with dysmenorrhea would be interested in CAM.

Traditional Chinese Medicine (TCM) based Acupuncture is a popular and common form of CAM with many women using acupuncture treatment for gynecological issues (22)

*Acupuncture as a complex intervention:*

Acupuncture, in common with many other Complementary and Alternative or non-pharmacological therapies, is a complex intervention (23-26). The Medical Research Council (MRC) defines complex interventions as “built up from a number of components, which may act both independently and interdependently” (27,28)

A typical acupuncture consultation includes but is not limited to: history taking, self care and lifestyle advice, TCM diagnosis and explanation, palpation of the pulse and other parts of the body, observation of the tongue, insertion and manipulation of needles, as well as common co-modalities such as moxibustion and cupping (23,29,30).

There is some evidence that the advice given during the acupuncture treatments and the way that the advice is delivered is an important component of the clinical outcome, however its role in outcomes in the treatment of dysmenorrhea is unclear (31-33).

*Acupuncture treatment of Dysmenorrhea*

The most recent systematic reviews have found conflicting results in the effectiveness of acupuncture in the treatment of dysmenorrhea (34).

One large scale pragmatic trial (35) showed a significant reduction in menstrual pain and an increase in quality of life in women undergoing acupuncture treatments over a three month period compared to women having only usual care. This trial did not document components of the treatment or frequency of treatment and therefore it is impossible to determine what components of the acupuncture treatment contributed towards the positive outcome. There is conflicting evidence in the literature on role of dosage and stimulation style in improving clinical outcomes of acupuncture treatment of dysmenorrhea. Chinese based literature shows very positive results with higher frequency of treatment (36-39) and with the usage of electroacupuncture (40-43). However these are tempered by poor methodological quality (44) and by acupuncture treatments that do not represent clinical practice in Australia and New Zealand (unpublished data from thesis) and other western countries (45).

In addition to measuring outcomes for symptoms of dysmenorrhea women who complete the trial will be invited to take part in semi-structured interviews that will examine the acceptability of the differing interventions, the importance of the explanations and advice given by the practitioners as well as their experience in the trial itself.

*Methodological challenges:*

Recent papers from CAM experts (46) acupuncture researchers (29,47)and acupuncture practitioners (45,48) all emphasise the vital need for research to reflect and inform clinical practice.

This trial uses a mixed methods approach with a pragmatic framework (49) that allows trial design to be informed by both survey and focus group data that has been collected as part of this thesis. This allows a randomised controlled trial to be developed that accurately reflects a clinically relevant setting. To achieve a balance between a tightly and loosely defined intervention is an important consideration in evaluating acupuncture as it is delivered in a community setting (25). A manualised protocol has been developed from the data from practitioner interviews and focus groups to help achieve this balance. This manualisation has been successfully used in previous acupuncture trials to ‘distill’ expert advice into a form that allows both individualisation and reproducibility (26,50,51). This manualisation will allow the exploration of individual contributions of frequency and stimulation style by reducing the 'noise' present in the trial while still staying faithful to the theoretical framework that underpins TCM acupuncture (23,24). In addition it is important that the study documentation and protocol conforms to the guidelines set out by Smith and colleagues (2011) that discuss the importance of reporting different components of the acupuncture treatment (52).

## 1.2 Study Rationale

This project will increase the evidence base on the effectiveness of treating primary dysmenorrhea with acupuncture based on a trial design that closely mimics clinical practice in Australia and New Zealand. The primary focus of this trial is the contribution of two theoretically important components of acupuncture ‘dosage’, frequency of treatment and type of needle stimulation, to the clinical outcomes. In addition the follow up interviews will allow women to have a qualitative ‘voice’ on their experiences as part of the trial.

# 2. Study Objectives

The primary aim of this project is to examine the effect of changing frequency and type of stimulation on the symptoms of primary dysmenorrhea.

The specific questions that this project address are:

1. Does using a manualised protocol based on current clinical experience provide a positive outcome with symptoms of primary dysmenorrhea?
2. What extent do differing frequencies of treatment and types of stimulation contribute to that effect?
3. Is acupuncture, especially in more frequent treatments, an acceptable intervention for participants ?
4. Does TCM acupuncture change study participants perceptions of health and illness with respect to dysmenorrhea ?
5. What other components of the acupuncture treatment are important to study participants ?

# 3. Study Design

A Phase III randomized controlled trial comparing 4 groups incorporating differing modes of stimulation and treatment frequency on pain and quality of life in women suffering from primary dysmenorrhea.

# 4. Study Population

## 4.1 Sample Size

68 women in total, 17 participants per study group

## 4.2 Recruitment

Local family planning clinics in Auckland and Wellington as well as Student health at local universities (University of Auckland and Victoria University,Wellington)

## 4.3 Inclusion Criteria

- Women with suspected or confirmed primary dysmenorrhea as defined by the following:
- Age 18-45 years
- Period pain started before the age of 18 OR Pain started > 18 but Ultrasound and Laparoscopy negative for secondary pathology.
- Pain greater than or equal to 3 out of 10 on a numeric rating scale during the first three days of menses for at least two of the past 3 menstrual cycles.
- Regular menstrual cycles (28 +/- 7 days) for the last three months.
- Understands spoken and written English
- Able to give informed consent

## 4.4 Exclusion Criteria

- Previous diagnosis of endometriosis or secondary dysmenorrhea.
- Abdominal Surgery in the previous 3 months
- Injectable or implant contraceptives (Depo provera, Jadelle, Murina ) within the last 3 months
- Oral Contraceptive usage started less than 3 months ago
- Chronic pain conditions ( >14 days per month with pain)
- Current mental health illness
- Neuropathic pain secondary to surgery
- Sterilisation

# 5. Participant selection and enrollment

## 5.1 Identifying participants

Recruitment:

A number of strategies will be used in progressive phases dependent on recruitment:

1. Posters will be placed in Family planning clinics and at Student health in local universities and 18-45 year old women presenting at these clinics with symptoms of period pain (dysmenorrhea) will be given an information sheet and contact details of the primary researcher.
2. Facebook advertisement and Facebook page promoting the study will be designed
3. If recruitment is still not sufficient at these sites then local PHO's such as Procare will be approached and GP's operating under the umbrella of these PHOs invited to assist in recruitment.

## 5.2 Screening for eligibility:

Initial screening will be undertaken over the phone by the researcher. Women who meet the eligibility criteria and are interested in joining the study will be met in person if they are located in Auckland or further information will be given via post or email if in Wellington. Prior to trial entry but post consent potential participants will be asked to fill in a menstrual pain diary that will be used to prevent recall error and validate that the participants do have maximum/peak pain greater than or equal to 3/10 and that this pain occurs just prior too, or in the first three days of menses. If these participants are eligible and are randomized then this data will become the baseline data.

## 5.3 Consenting participants

For those that consent and have an eligible menstrual pain diary a trial entry form will record baseline clinical data as well as demographic characteristics. Randomisation will occur after trial entry form completed.

## 5.4 Ineligible and non-recruited participants

Women who do not meet the initial eligibility criteria or whose menstrual pain diaries do not meet eligibility criteria will be logged. Women who decline to participate after initial contact will also be logged, with a reason for declining (if given).

## 5.5 Randomisation

5.5.1 Randomisation

Randomisation will be undertaken by using the secure internet website Sealed Envelope (Sealedenvelope.com). This will produced a randomization schedule with variable blocking.

5.5.2 Treatment allocation

Randomization will be into one of four study groups:

High Frequency – Manual stimulation: Women randomly allocated to this group will receive 3 acupuncture treatments in the 7 days prior to menses and 1 acupuncture treatment in the first 3 days of menses. Stimulation will be provided by manual acupuncture, achieving DeQi upon needle insertion. Subsequent stimulation based on practitioners differential diagnosis. Needles will be retained for 20-30 minutes

High Frequency – Electro acupuncture stimulation: Women randomly allocated to this group will receive 3 acupuncture treatments in the 7 days prior to menses and 1 acupuncture treatment in the first 3 days of menses. Stimulation will be provided by Electro-Acupuncture, achieving DeQi upon needle insertion. Subsequent stimulation will be using a 2/100Hz waveform for 20-30 minutes.

Low Frequency – Manual Stimulation: Women randomly allocated to this group will receive 3 acupuncture treatments in the 3 weeks prior to menses (1/week) and 1 acupuncture treatment in the first 3 days of menses. Stimulation will be provided by manual acupuncture, achieving DeQi upon needle insertion. Subsequent stimulation based on practitioners differential diagnosis.Needles will be retained for 20-30 minutes

Low Frequency – Electro acupuncture stimulation: Women randomly allocated to this group will receive 3 acupuncture treatments in the 3 weeks prior to menses (1/week) and 1 acupuncture treatment in the first 3 days of menses. Stimulation will be provided by Electro-Acupuncture, achieving DeQi upon needle insertion.Subsequent stimulation will be using a 2/100Hz waveform for 20-30 minutes.

5.5.3: Withdrawal procedures

If a participant wishes to withdraw from the trial they will be immediately removed from treatment. The most recent menstrual pain diary will be used and they will be requested to complete an SF-36.

If a participant withdraws during intervention they will not be replaced however if they withdraw post randomization but prior to the first treatment they will be replaced.

# 6. Investigational therapy and controls

## 6.1 Acupuncture

A total of 12 treatments over 3 menstrual cycles will be administered according to the frequency specified in the subjects’ group allocation. The study acupuncturists will include the primary investigator (Mike Armour) and all study acupuncturists will be experienced, with a minimum of a Bachelors level qualification in Acupuncture and will hold a current annual practicing certificate with a relevant professional acupuncture body. Practitioners will be located in Auckland and Wellington, New Zealand. Mike Armour will also be involved in training and quality control of other study acupuncturists.

Following the first phase of this mixed methods PhD thesis a trial protocol has been developed based around using a manualised version (50) of standard acupuncture treatments which has been designed in consultation with experienced practitioners through interviews and focus groups.

This manualisation allows considerable flexibility, in order that the treatment can match patient variability. The protocol is not too broad, such that “anything goes” as this would mean that it would be more difficult to attribute any impact of treatment to the different interventions we are using, frequency and stimulation of treatment. The protocol is also not too narrow, as we want the treatments to reflect how acupuncturists normally work in clinical practice. This is an important consideration in designing a clinically relevant intervention (25).

The rationale for the individualization of treatments is based on the TCM principle of “*Yi bing tong zhi, tong bing yi zhi*” translated to mean “Different diseases, same treatment. Same disease, different treatment” (pp2) (53). To allow for this important component of TCM’s theoretical framework to be preserved, clinicians will take a history and give a TCM diagnosis during the first session. This will be guided by key diagnostic factors that have been stressed by experienced clinicians. Once a TCM diagnosis has been ascertained then practitioners will have flexibility in their point selection for this pattern. Each pattern has a selection of ‘vital’ points that have been identified by expert practitioners as being vital to the treatment outcome for each pattern. At least 3 of these ‘vital’ points will need to be used in each treatment session. Practitioners will then be able to choose from an optional list of points for each pattern that can be used as desired. Other common co-interventions such as moxibustion, a warming therapy often used in the treatment of gyanecological complaints due to ‘cold’, are also allowed (54). Herbal medicine or additional supplementation is not to be provided by practitioners during the trial. Each subsequent treatment session will allow for a confirmation or update on the TCM diagnosis and therefore point selection may be varied from treatment to treatment.

Acupuncture points will be needled bilaterally except those where only one side is used clinically such as opening extraordinary vessels such as the Chong Mai (54). Stimulation parameters are detailed in the group assignments. Point location and needling depth will be as specified in *A Manual of Acupuncture* (55). Practitioners will maintain logbooks detailing all aspects of treatment provided (including co-interventions such as moxibustion). Single use, stainless steel needles of varying gauge (.20 x 30mm or .25 x 40mm), dependent on body shape, will be used.

After the conclusion of the 3 menstrual cycles there will be an approximately one-month follow up period ending on the completion of the subjects subsequent menstrual period.

## 6.2 Control group

There is no control group in this study as it is designed to examine the differing contribution of different acupuncture components to the clinical outcome.

## 6.3 Prior and Concomitant Medications

### 6.3.1 Permitted medications

Subjects will continue with all prescribed medication and can use analgesics as needed for pain relief.

# 7. Study Assessments

## 7.1 Safety Assessments

At each treatment session the treating acupuncturist will ask the participants if they have had any adverse events or reactions after the last treatment. Any adverse events or reactions that are thought to be causally associated with the intervention will be recorded in the practitioners log book and reported to the primary investigator Mike Armour. Any adverse events reported to the PI will be discussed with the supervisory panel and assessed for clinical significance. All adverse events reported during the duration of the trial (including follow-up) will be recorded under adverse events as part of the case report form.

Participants during the interviews will also be asked about any adverse events or unpleasant experiences during the trial.

Minor adverse events for acupuncture and moxibustion are expected to occur at a rate of around 1.3 per 1,000 treatments (56) and mostly include local pain on needling, mild bruising and bleeding (57). The risk of burns from moxibustion is very rare (56-58).

## 7.2 Study assessments

The menstrual pain diary will be used to assess primary and secondary endpoints at baseline and at the end of every menstrual period during the 3 month study period. Follow-up pain diaries will also be collected at 1, 6 and 12 months post trial completion.

The SF-36 will be assessed at baseline and then at 1 month post trial completion.

Patient satisfaction with the trial will be assessed at the end of the trial with an exit questionnaire given after the final acupuncture treatment.

# 8. Data Collection

The primary investigator, Mike Armour, will be responsible for collection of these outcomes at baseline, after each subsequent menstrual period during the trial phase and then again at one, six and twelve months post trial completion.

Baseline data includes age, ethnicity, height, weight, age of menarche, onset of dysmenorrhea, length of menstrual cycle, length of menstrual period, smoking status, alcohol consumption, baseline analgesic usage and self reported dysmenorrhea symptoms and severity. A 5 point Likert scale will assess prior belief in acupuncture’s effectiveness.

*Primary outcome measure:*

Numerical rating scale (NRS) for pain. Each subject will keep a menstrual diary provided by the researcher. This will note the day of menses, average and maximum pain per day on an 11 point NRS with 0 being no pain and 10 being the worst pain imaginable. NRS is used in preference to VAS as it is comparable (59) but has a higher compliance rate, is easier to use and fits the compact format of a menstrual diary (60). The primary endpoint is a 20% difference in NRS rated average pain severity between groups.

*Secondary outcome measures:*

Secondary outcome measures which will be captured by the menstrual diary are duration of menstrual pain (hours), menstrual characteristics (flow, clots), painkillers taken (type, dosage, number of doses), non pharmacological interventions (heat,rest) and secondary symptoms of dysmenorrhea (headache, vomiting etc).

HRQoL will be recorded by the Short Form Health Survey (SF-36) at two time points; baseline and at the one month follow-up. The SF-36 has been shown to be valid and reliable across a range of populations (61,62) and has shown suitability for use in dysmenorrhea (63).

An exit questionnaire will be used to determine patient satisfaction with the different interventions and examine which components of the consultation and treatment sessions were important.

*Quality assurance:*

Quality assurance measures on needling technique and point selection will be carried out on practitioners during the study period by using an independent acupuncturist to check point selection and location at least once for each practitioner. Needling technique will be measured using a newly validated tool, the AcuSensor (64).

*In-depth interviews:*

These questions will focus on the participants experience in the trial and includes discussion on the therapeutic relationship, beneficial and negative aspects of the trial, explanation of TCM therapeutics and the provision of self-care/self help advice. These interviews will be recorded via a digital recorder.

# 9. Statistics and Data Analysis

## 9.1 Sample Size calculation:

This trial is designed to demonstrate a 20% difference in average pain intensity between groups as measured by the Numeric Rating Scale (NRS). Based on previous study data (65) a standard deviation of 1.3 units on a pain scale was used. An alpha of .05 and power of 90% gives a total sample size of 60 women. Previous studies (35,65) have had relatively low loss to follow-up (~10%) therefore a sample size of 68 women will be used to account for the predicted loss to follow-up of 10%.

## 9.2 Proposed Analysis:

For the data from the clinical trial a range of data analysis techniques will be undertaken including simple descriptive statistics; two way repeated measures ANOVA for NRS pain scores and SF-36 scores. Regression analysis with GLM will be used to examine factors influencing NRS scores, analgesic intake, secondary symptoms and non-pharmacological therapies. Non-parametric data such as analgesic intake, presence of secondary symptoms and usage of non-pharmacological therapies will be analysed by Mann-Whitney U-test.

Data from the in-depth interviews will be transcribed verbatim from the digital recording by the researcher. NVIVO will be used to manage the transcribed data and digital recordings. Thematic analysis will be used to identify emergent themes from the data.

# 10. Monitoring and quality assurance

## 10.1 Project management and trial management group

The primary investigator, Mike Armour, will have regular meetings with his supervisory panel and will discuss recruitment, compliance, retention and follow up as well as the implementation of the overall trial protocol. These meetings will also discuss any adverse event data reported by study acupuncturists.

Mike Armour will also have regular email/phone conversations with other study acupuncturists to discuss any issues with recruitment, compliance, retention and follow up as well as the implementation of the overall trial protocol. In addition to this quality control will be undertaken at several points during the trial with each study acupuncturist being assessed at least once. Prior to the implementation of the protocol all study acupuncturists will be trained by the Primary Investigator and the AcuSensor device will be used to standardize needling technique to ensure that similar quality of stimulation is delivered by all study acupuncturists.

There is no data monitoring committee for this study.

# 11. Good Clinical Practice Module

## 11.1 Ethical conduct of the study

The research has been submitted to both the UWS Human Research Ethics committee and the HDEC for approval.

## 11.2 Investigator responsibilities

11.2.1 Informed consent

All participants will be required to give informed consent prior to being issued with the menstrual pain diary. Informed consent will be undertaken either face to face with Mike Armour or via post.

11.2.2 Emergency contact with Investigators

All participants will be given emergency contact numbers of both the study acupuncturists and the primary investigator Mike Armour

11.2.3 Investigator indemnification

Following ethical approval, clinical trial insurance will be registered with the University

11.2.4 Study site staff

Mike Armour

11.2.5 Data Recording

All data will be entered electronically into a secure database with offsite backups at Centre for Complementary Medicine Research at The University of Western Sydney

11.2.6 Confidentiality

All data is confidential. All study participants will be allocated a Study ID

11.2.7 Data protection

All electronic files will be password protected. All paper files will be kept in locked filing cabinets at the home of the primary investigator, Mike Armour

# 12. Reporting, Publication and Notification of Results

## 12.1 Authorship policy

The Primary investigator and supervisory panel will be authors of the main manuscript.

## 12.2 Publication

The study results will form part of the primary investigators PhD thesis. In addition these results will be presented at conferences and published in appropriate journals.

# 13. References

1. Proctor M, Farquhar C. Diagnosis and management of dysmenorrhoea. BMJ: British Medical Journal 2006;332(7550):1134.

2. Wong CL, Farquhar C, Roberts H, Proctor M. Oral contraceptive pill for primary dysmenorrhoea. Cochrane Database Syst Rev 2009(4):CD002120.

3. Coco AS. Primary dysmenorrhea. Am Fam Physician 1999, Aug;60(2):489-96.

4. Bettendorf B, Shay S, Tu F. Dysmenorrhea: Contemporary perspectives. Obstet Gynecol Surv 2008, Sep;63(9):597-603.

5. Zahradnik HP, Hanjalic-Beck A, Groth K. Nonsteroidal anti-inflammatory drugs and hormonal contraceptives for pain relief from dysmenorrhea: A review. Contraception 2010, Mar;81(3):185-96.

6. Latthe P, Mignini L, Gray R, Hills R, Khan K. Factors predisposing women to chronic pelvic pain: Systematic review. BMJ 2006;332(7544):749-55.

7. French L. Dysmenorrhea in adolescents: Diagnosis and treatment. Paediatr Drugs 2008;10(1):1-7.

8. Hillen TI, Grbavac SL, Johnston PJ, Straton JA, Keogh JM. Primary dysmenorrhea in young western australian women: Prevalence, impact, and knowledge of treatment. J Adolesc Health 1999, Jul;25(1):40-5.

9. Dawood MY. Ibuprofen and dysmenorrhea. Am J Med 1984, Jul 13;77(1A):87-94.

10. Marjoribanks J, Proctor M, Farquhar C, Derks RS. Nonsteroidal anti-inflammatory drugs for dysmenorrhoea. Cochrane Database Syst Rev 2010(1):CD001751.

11. Griffin MR. Epidemiology of nonsteroidal anti-inflammatory drug-associated gastrointestinal injury. Am J Med 1998, Mar 30;104(3A):23S-29S; discussion 41S-42S.

12. Trelle S, Reichenbach S, Wandel S, Hildebrand P, Tschannen B, Villiger PM, et al. Cardiovascular safety of non-steroidal anti-inflammatory drugs: Network meta-analysis. BMJ 2011;342:c7086.

13. Shi S, Klotz U. Clinical use and pharmacological properties of selective COX-2 inhibitors. Eur J Clin Pharmacol 2008, Mar;64(3):233-52.

14. Roumie CL, Choma NN, Kaltenbach L, Mitchel EF, Arbogast PG, Griffin MR. Non-aspirin nsaids, cyclooxygenase-2 inhibitors and risk for cardiovascular events-stroke, acute myocardial infarction, and death from coronary heart disease. Pharmacoepidemiol Drug Saf 2009, Nov;18(11):1053-63.

15. Howard, Perry, Carter, El-Minawi. Pelvic pain diagnosis and management. Philadelphia: Lippincot Williams and Wilkins; 2000.

16. Dawood MY. Dysmenorrhea. Clin Obstet Gynecol 1990, Mar;33(1):168-78.

17. Pledger MJ, Cumming JN, Burnette M. Health service use amongst users of complementary and alternative medicine. N Z Med J 2010, Apr 9;123(1312):26-35.

18. Shorofi SA. Complementary and alternative medicine (CAM) among hospitalised patients: Reported use of CAM and reasons for use, CAM preferred during hospitalisation, and the socio-demographic determinants of CAM users. Complement Ther Clin Pract 2011, Nov;17(4):199-205.

19. Thomson P, Jones J, Evans JM, Leslie SL. Factors influencing the use of complementary and alternative medicine and whether patients inform their primary care physician. Complement Ther Med 2012, Feb;20(1-2):45-53.

20. Xue CC, Zhang AL, Lin V, Da Costa C, Story DF. Complementary and alternative medicine use in australia: A national population-based survey. J Altern Complement Med 2007;13(6):643-50.

21. Yates KM, Armour MJ, Pena A. Complementary therapy use amongst emergency medicine patients. Complement Ther Med 2009, Aug;17(4):224-8.

22. Hopton AK, Curnoe S, Kanaan M, Macpherson H. Acupuncture in practice: Mapping the providers, the patients and the settings in a national cross-sectional survey. BMJ Open 2012;2(1):e000456.

23. Paterson C, Britten N. Acupuncture as a complex intervention: A holistic model. J Altern Complement Med 2004, Oct;10(5):791-801.

24. Paterson C, Dieppe P. Characteristic and incidental (placebo) effects in complex interventions such as acupuncture. BMJ 2005, May 21;330(7501):1202-5.

25. MacPherson H, Schroer S. Acupuncture as a complex intervention for depression: A consensus method to develop a standardised treatment protocol for a randomised controlled trial. Complement Ther Med 2007, Jun;15(2):92-100.

26. Schnyer RN, Iuliano D, Kay J, Shields M, Wayne P. Development of protocols for randomized sham-controlled trials of complex treatment interventions: Japanese acupuncture for endometriosis-related pelvic pain. J Altern Complement Med 2008, Jun;14(5):515-22.

27. Campbell M, Fitzpatrick R, Haines A, Kinmonth AL, Sandercock P, Spiegelhalter D, Tyrer P. Framework for design and evaluation of complex interventions to improve health. BMJ 2000;321(7262):694-6.

28. Craig P, Dieppe P, Macintyre S, Michie S, Nazareth I, Petticrew M, Medical Research Council Guidance. Developing and evaluating complex interventions: The new medical research council guidance. BMJ 2008;337:a1655.

29. Langevin HM, Wayne PM, Macpherson H, Schnyer R, Milley RM, Napadow V, et al. Paradoxes in acupuncture research: Strategies for moving forward. Evid Based Complement Alternat Med 2011;2011:180805.

30. MacPherson H, Thorpe L, Thomas K. Beyond needling--therapeutic processes in acupuncture care: A qualitative study nested within a low-back pain trial. J Altern Complement Med 2006, Nov;12(9):873-80.

31. de Lacey S, Smith CA, Paterson C. Building resilience: A preliminary exploration of women's perceptions of the use of acupuncture as an adjunct to in vitro fertilisation. BMC Complement Altern Med 2009;9:50.

32. Paterson C, Evans M, Bertschlinger R, Chapman R, Norton R, Robinson J. Communication about self-care in traditional acupuncture consultations: The co-construction of individualised support and advice. Patient Educ Couns 2012, Mar 23.

33. Evans M, Paterson C, Wye L, Chapman R, Robinson J, Norton R, Bertschinger R. Lifestyle and self-care advice within traditional acupuncture consultations: A qualitative observational study nested in a co-operative inquiry. J Altern Complement Med 2011, Jun;17(6):519-29.

34. Smith CA, Zhu X, He L, Song J. Acupuncture for primary dysmenorrhoea. Cochrane Database Syst Rev 2011(1):CD007854.

35. Witt CM, Reinhold T, Brinkhaus B, Roll S, Jena S, Willich SN. Acupuncture in patients with dysmenorrhea: A randomized study on clinical effectiveness and cost-effectiveness in usual care. Am J Obstet Gynecol 2008, Feb;198(2):166.e1-8.

36. Xiong J, Liu F, Zhang MM, Wang W, Huang GY. De-qi, not psychological factors, determines the therapeutic efficacy of acupuncture treatment for primary dysmenorrhea. Chin J Integr Med 2012, Jan;18(1):7-15.

37. Yu YP, Ma LX, Ma YX, Ma YX, Liu YQ, Liu CZ, et al. Immediate effect of acupuncture at sanyinjiao (SP6) and xuanzhong (GB39) on uterine arterial blood flow in primary dysmenorrhea. J Altern Complement Med 2010, Oct;16(10):1073-8.

38. Jiang L. Clinical experience for the treatment of 34 cases of primary dysmenorrhoea. Journal of Emergency TCM 2007;16:620-1.

39. Li CH, Wang Y, Ge X. Clinical observation of the use of acupuncture on 4 gate points for primary dysmenorrhoea. Journal Chinese Acupuncture and Moxibustion 2008;28:187-90.

40. Shi GX, Liu CZ, Zhu J, Guan LP, Wang DJ, Wu MM. Effects of acupuncture at sanyinjiao (SP6) on prostaglandin levels in primary dysmenorrhea patients. Clin J Pain 2011;27(3):258-61.

41. Zhi L. Randomised controlled study on superficial needling for treatment of primary dysmenorrhea. Chinese Acupuncture and Moxibustion 2007;27(1):18-21.

42. Ma YX, Ma LX, Liu XL, Ma YX, Lv K, Wang D, et al. A comparative study on the immediate effects of electroacupuncture at sanyinjiao (SP6), xuanzhong (GB39) and a non-meridian point, on menstrual pain and uterine arterial blood flow, in primary dysmenorrhea patients. Pain Med 2010, Oct;11(10):1564-75.

43. Liu CZ, Xie JP, Wang LP, Zheng YY, Ma ZB, Yang H, et al. Immediate analgesia effect of single point acupuncture in primary dysmenorrhea: A randomized controlled trial. Pain Med 2011, Feb;12(2):300-7.

44. Cho SH, Hwang EW. Acupuncture for primary dysmenorrhoea: A systematic review. BJOG 2010, Apr;117(5):509-21.

45. Kaptchuk TJ, Chen KJ, Song J. Recent clinical trials of acupuncture in the west: Responses from the practitioners. Chin J Integr Med 2010, Jun;16(3):197-203.

46. Fønnebø V, Grimsgaard S, Walach H, Ritenbaugh C, Norheim AJ, MacPherson H, et al. Researching complementary and alternative treatments--the gatekeepers are not at home. BMC Med Res Methodol 2007;7:7.

47. Wayne PM, Hammerschlag R, Langevin HM, Napadow V, Park JJ, Schnyer RN. Resolving paradoxes in acupuncture research: A roundtable discussion. J Altern Complement Med 2009, Sep;15(9):1039-44.

48. Robinson N, Lorenc A, Ding W, Jia J, Bovey M, Wang XM. Exploring practice characteristics and research priorities of practitioners of traditional acupuncture in china and the EU-A survey. J Ethnopharmacol 2012, Feb 7.

49. Creswell JW, Plano Clark VL. Designing and conducting mixed methods research. 2, illustrated ed. Los Angeles: Sage Publications; 2010.

50. Schnyer RN, Allen JJ. Bridging the gap in complementary and alternative medicine research: Manualization as a means of promoting standardization and flexibility of treatment in clinical trials of acupuncture. J Altern Complement Med 2002, Oct;8(5):623-34.

51. Schnyer RN, Wayne PM, Kaptchuk TJ, Cheng X, Zhang Z, Stason WB. Standardization of individualized treatments in a randomized controlled trial of acupuncture for stroke rehabilitation. J Altern Complement Med 2006, Mar;12(2):106-9.

52. Smith CA, Zaslawski CJ, Zheng Z, Cobbin D, Cochrane S, Lenon GB, et al. Development of an instrument to assess the quality of acupuncture: Results from a delphi process. J Altern Complement Med 2011, May;17(5):441-52.

53. Flaws B, Sionneau P. The treatment of modern western medical diseases with chinese medicine : A textbook & clinical manual. Boulder, CO: Blue Poppy Enterprises; 2001.

54. Maciocia G, Kapthcuk TJ. Obstetrics and gynecology in chinese medicine 2011:1092 Available from: http://site.ebrary.com/id/10537500.

55. Deadman P, Al-Khafaji M, Baker K. A manual of acupuncture. 2, revised ed. Hove, East Sussex, England; Vista, Calif.: Journal of Chinese Medicine; 1998.

56. MacPherson H, Thomas K, Walters S, Fitter M. A prospective survey of adverse events and treatment reactions following 34,000 consultations with professional acupuncturists. Acupunct Med 2001, Dec;19(2):93-102.

57. Witt CM, Pach D, Brinkhaus B, Wruck K, Tag B, Mank S, Willich SN. Safety of acupuncture: Results of a prospective observational study with 229,230 patients and introduction of a medical information and consent form. Forsch Komplementmed 2009, Apr;16(2):91-7.

58. Park JE, Sul JU, Kang K, Shin BC, Hong KE, Choi SM. The effectiveness of moxibustion for the treatment of functional constipation: A randomized, sham-controlled, patient blinded, pilot clinical trial. BMC Complement Altern Med 2011;11:124.

59. Bijur PE, Latimer CT, Gallagher EJ. Validation of a verbally administered numerical rating scale of acute pain for use in the emergency department. Acad Emerg Med 2003, Apr;10(4):390-2.

60. Hjermstad MJ, Fayers PM, Haugen DF, Caraceni A, Hanks GW, Loge JH, et al. Studies comparing numerical rating scales, verbal rating scales, and visual analogue scales for assessment of pain intensity in adults: A systematic literature review. J Pain Symptom Manage 2011, Jun;41(6):1073-93.

61. Brazier JE, Harper R, Jones NM, O'Cathain A, Thomas KJ, Usherwood T, Westlake L. Validating the SF-36 health survey questionnaire: New outcome measure for primary care. BMJ 1992, Jul 18;305(6846):160-4.

62. Walters SJ, Munro JF, Brazier JE. Using the SF-36 with older adults: A cross-sectional community-based survey. Age Ageing 2001, Jul;30(4):337-43.

63. Unsal A, Ayranci U, Tozun M, Arslan G, Calik E. Prevalence of dysmenorrhea and its effect on quality of life among a group of female university students. Ups J Med Sci 2010, May;115(2):138-45.

64. Davis RT, Churchill DL, Badger GJ, Dunn J, Langevin HM. A new method for quantifying the needling component of acupuncture treatments. Acupunct Med 2012, Mar 16.

65. Smith CA, Crowther CA, Petrucco O, Beilby J, Dent H. Acupuncture to treat primary dysmenorrhea in women: A randomized controlled trial. Evid Based Complement Alternat Med 2011;2011:612464.
